# Computational Workflow to Unravel the Structural Dynamics of Supramolecular Metallacages in Solution

**DOI:** 10.1021/acs.jctc.5c01465

**Published:** 2025-11-20

**Authors:** Julia A. Stebani, Iñigo Iribarren Aguirre, Gohar A. Siddiqui, Darren Wragg, Alessio Gagliardi, Angela Casini

**Affiliations:** † Medicinal and Bioinorganic Chemistry, Department of Chemistry, School of Natural Sciences, 9184 Technical University of Munich, Lichtenbergstr. 4, 85748 Garching b. München, Germany; ‡ Simulation of Nanosystems for Energy Conversion, Department of Electrical Engineering & Atomistic Modeling Center (AMC), School of Computation, Information and Technology, 9184 Technical University of Munich, Hans-Piloty-Str. 1, 85748 Garching b. München, Germany; § Munich Data Science Institute (MDSI), 9184 Technical University of Munich, Walther-Von-Dyck Str. 10, 85748 Garching b. München, Germany

## Abstract

The structural dynamics of self-assembled metallacages is important because it determines their function and stability in different applications involving encapsulation and release of a guest molecule. We present here an integrated computational workflow to study the dynamic behavior of selected [Pd_2_L_4_]^4+^ metallacages in explicit solvents (water and DMSO) and benchmark them for future *in silico* investigations of their host–guest chemistry, pivotal to their application as drug delivery systems. Two different pathways for the Molecular Dynamics (MD) simulations of the systems are explored, namely classical force field (FF) and Machine Learning Interatomic Potentials (MLIPs), to assess the conformational changes of two cage systems in solution, enabling evaluation of the performance vs computational cost for both methodologies. The proposed workflow offers a versatile framework to computationally assess the structural dynamics of supramolecular systems in solution, effectively bridging the gap between quantum-level accuracy and the temporal and spatial scales needed for simulations of different functional applications.

## Graphical abstract

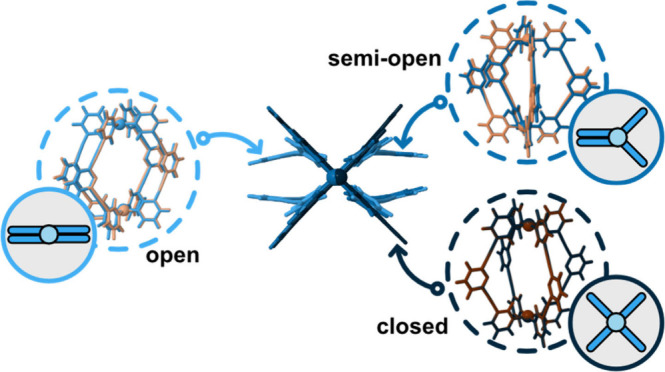

## Introduction

Porous metal–organic supramolecular coordination cages (MCgs) hold promise for a plethora of applications,^1^ including catalysis,^2–5^ sensing,^6^ biomedical imaging^7^ and drug delivery,^8–10^ thanks to their design flexibility and host–guest chemistry, enabling them to encapsulate small molecules into their cavity. MCgs are discrete molecular arrangements composed of metal nodes bound to organic ligands brought together via coordination-driven self-assembly (CDSA).^11^ Among the various MCgs families, we recently focused our attention on lantern-shaped cationic [Pd_2_L_4_]^4+^ cages (L = bitopic monodentate N-donor ligand, Figure 1), exploring them for different potential biomedical uses. For example, MCgs were proven effective as targeted drug delivery systems for the anticancer drug cisplatin,^12^ as well as for the delivery of radioactive ^99m^TcO_4_^–^ for single photon computed tomography (SPECT) applications.^13^

**Figure 1:**
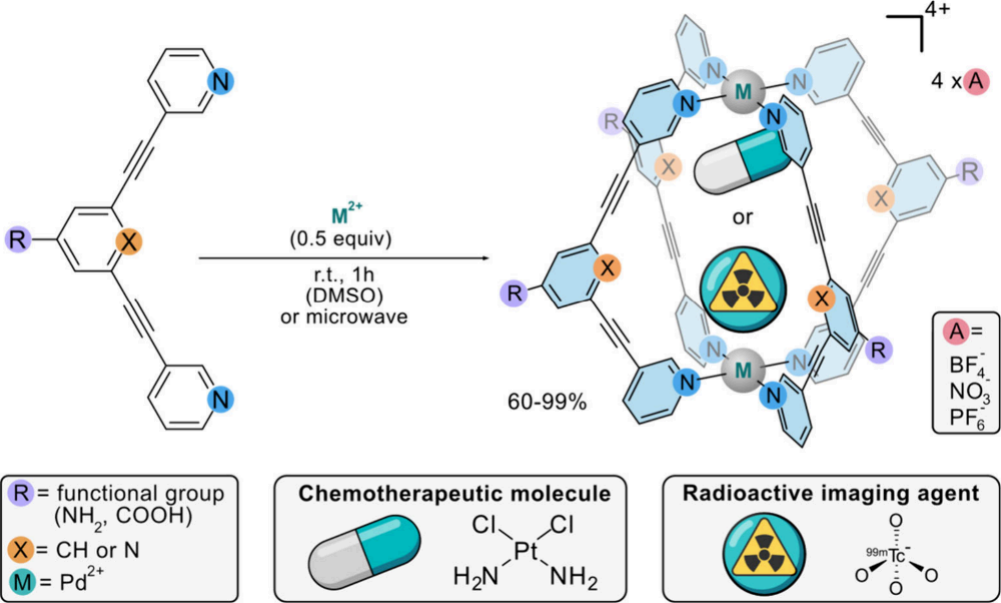
Schematic representation of the synthetic approach to achieve [Pd_2_L_4_]^4+^ MCgs by CDSA and their use for small molecule encapsulation, e.g., therapeutic anticancer drugs (cisplatin) or imaging agents (^99m^TcO_4_^–^). Ligands can incorporate different *exo*- and/or *endo*-functionalities. The cationic MCg requires counteranions, such as PF_6_, BF_4_^–^, or NO_3_^–^, among others.

To facilitate the MCgs design and optimize their host–guest chemistry, understanding the established noncovalent interactions of guest compounds, including solvent molecules, within the cage cavity at the atomic level is essential. It has been shown that the replacement of high-energy water in MCg cavities by guest molecules is the key enthalpic driving force for cage/cargo adduct formation,^14,15^ which makes the characterization of the solvent-MCgs interactions of utmost importance. Computational methods can provide insights into these interactions at an atomistic level, beyond the prediction of synthetically viable structures, and enable *in silico* screening. Today, several strategies are available to model noncovalent interactions, such as classical Molecular Dynamics (MD) or Monte Carlo (MC) methods, often combined for large systems with hybrid *ab initio* Quantum Mechanical and classical Molecular Mechanics (QM/MM) approaches to maximize the accuracy at an affordable cost, whereby the interaction is modeled at the QM level and the environment described using force field (FF)-based approaches.^16–18^ Thus, QM/MM simulations by definition use hybrid potentials to be used in energy minimizations.^19^ A combination of approaches is typically required rather than relying solely on one approach. While computational design and screening workflows have long been known as powerful tools in materials discovery, their application to MCgs is more recent.^17,20^ Nevertheless, examples of structure generation, topology prediction, self-assembly process prediction, guest binding affinity calculation, and confinement effects estimation on catalytic and sensing capabilities have been reported by many groups. Some examples related to [Pd_2_L_4_]^4+^ cages have also appeared and proven to be invaluable for supporting experimental findings.^5,21–23^ For example, Duarte and co-workers investigated the flexibility of [Pd_2_L_4_]^4+^ cages during MD simulations and the influence of the measured conformational distortions on guest binding affinity.^21^ Moreover, they showed how structural differences in [Pd_2_L_4_]^4+^ cavities impact the activity of the MCgs as supramolecular catalytic vessels for mild chlorinating agents.^24^ Tarzia et al. developed a joint computational and experimental workflow for the tailored synthesis of low-symmetry [Pd_2_L_4_]^4+^ cages with anisotropic cavities toward improved specificity for guest binding.^25^ Schäfer and co-workers have analyzed the encapsulation process in several MCgs.^26^ Further, Clever and co-workers evaluated the contribution of dispersive host–guest interactions to binding in Pd-cages from electronic structure calculations.^27^

It should be noted that, other than via enhanced sampling methods (e.g., metadynamics^28,29^ ), interactive mechanisms and even binding events can only be evaluated by combining *ab initio* approaches that treat relevant atom groups quantum mechanically for improved accuracy while handling surrounding atom groups, and especially solvent molecules, with classical force fields. These QM/MM calculations can account for covalent bond breaking and bond-forming reactions without the limitation of only a small number of semiempirically established parameters defined in a force field.^16^ While *ab initio* MD methods allow for the study of condensed systems, including their electronic structure, the computational cost for such calculations is high and limited in size.^30–32^ Current efforts have focused on including electronic structure data into MD simulations by the introduction of Machine Learning Interatomic Potentials (MLIPs).^30,33,34^ Here, cumbersome parametrization is overcome by training on high-fidelity QM data that includes electronic effects such as bond breaking/formation and polarization. The advantages of MLIPs are an improved accuracy versus classical FFs, while offering lower computational cost compared to *ab initio* calculations, and thus, enabling simulations of longer time scales or larger molecules than conventional QM methods.^33^ In addition, unlike empirical FFs that require predefined functions, MLIPs can extract complex patterns from large data sets without such constraints. For example, Wang et al. developed a biomolecular dynamics system AI^2^BMD, with the use of a MLIP that can model large biomolecules at the atomic level with the precision of *ab initio* methods.^35^ In addition, Zhang et al. demonstrated a strategy for modeling chemical reactions in explicit solvents using MLIPs for the investigation of solvent–solute interactions.^36^ These examples highlight the potential of MLIPs for improved simulations of noncovalent supramolecular assemblies and interactions. Nevertheless, to the best of our knowledge, despite their potential, there are no examples of their application to MCgs.

In this study, we establish a parametrization workflow to investigate the dynamic behavior of lantern-shaped [Pd_2_L_4_]^4+^ cages in different solvents, with the long-term aim of benchmarking them for future application as drug delivery systems. Our primary objectives are as follows:

1. Evaluate how different charge fitting models influence the electrostatic interactions and, consequently, the structural integrity and cavity formation of the cages in explicit solvent (water and DMSO) - properties that are pivotal for future studies of small-molecule encapsulation.
2. Provide a workflow for both classical FFs and machine learning interatomic potentials (MLIPs), based on an equivariant neural network architecture (NequIP^34^), trained on electronic structure data.
3. Provide a comparison between adapting the FF vs the MLIP route to MD simulations in terms of accuracy and computational cost.

We combine these objectives in a powerful and versatile workflow (Figure 2) that integrates *ab initio* QM calculations, classical molecular mechanics, and MLIP-based molecular dynamics simulations to provide a robust framework for modeling MCgs in solution.

**Figure 2:**
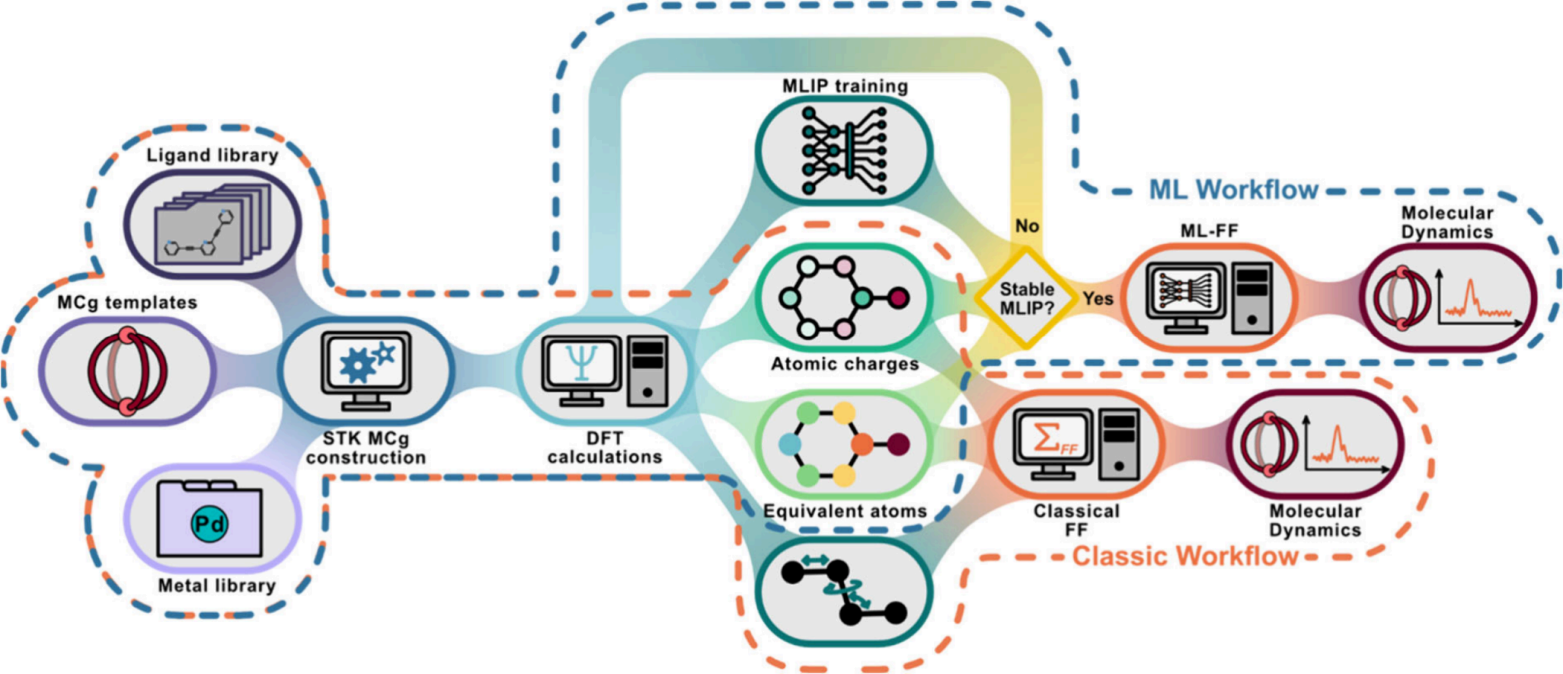
Schematic workflow of construction of force field parameters and charges for MD simulations, with splitting into two branches: classical AMBER force field construction and testing for two MCg analogs for standard (orange) and Machine Learning Interatomic Potential (MLIP)-based MD simulations (blue).

## Results and Discussion

To enable accurate modeling of [Pd_2_L_4_]^4+^ MCgs and to confirm their structural stability during molecular dynamics (MD) simulations in explicit solvent, we established a two-branched computational workflow (Figure 2) which consists of one common process branch for establishing the cage system and then, splitting into (*i*) classical or (*ii*) ML-assisted approaches, respectively, for the testing and comparison of the methods. Thus, we selected two representative [Pd_2_L_4_]^4+^ cage structures featuring either a hydrophobic or a hydrophilic cavity, namely *endo*-**C** (L = 1,3-bis­(pyridin-3-ylethynyl)­benzene) and *endo*-**N** (L = 2,6-bis­(pyridin-3-ylethynyl)­pyridine), respectively (Figure 1). The *endo*-**N** modification offers free lone pairs as hydrogen bond acceptor sites for small molecules encapsulated in the cavity, such as cisplatin.^12,37^ Detailed procedures and results are provided below, while the comprehensive step-by-step methodology is outlined in the Supporting Information. Simulations were carried out in both water and dimethyl sulfoxide (DMSO), the latter commonly used to enhance the solubility of metal–organic cages (MCgs).

The workflow begins with the MCgs’ construction and parameter optimization using *ab initio* Density Functional Theory (DFT) and the consecutive establishment of different generations (1st, 2nd, and 3rd generation) of a classical AMBER force field, which were used for classical MD simulations. The 2nd and 3rd generation FFs comprise parameter improvements made on the respective previous generation. A detailed description of the construction of force field parameters for each generation can be found in the SI.

For an accurate description of the MCgs’ dynamics in solvent, the assignment of nonbonded parameters is of high importance. MD generally accounts for the potential energy of a system as a function of bonded and nonbonded terms, where bonded terms are usually empirically established through experiments or high-level DFT calculations, and are supplied in the force field. Nonbonded interactions are considered by van der Waals forces through the application of Lennard-Jones, or similar potentials, as well as electrostatic interactions that are described by a Coulomb potential as a function of the atomic partial charges *q*_*i*_.^38^ The assignment of those partial charges is a crucial task during force field parametrization, as they are handled as static fixed-points and thus, will not be updated during the course of the simulation. Moreover, no experimental procedure is available for the verification of computationally assigned partial charges.^39^ In our study, in parallel to the determination of the bonded parameters, different electrostatic charge fitting models were applied for atomic partial charge assignment for nonbonded parameter determination. The methods for partial charge assignment can be divided into two families, i.e., partitioning of electron density according to contributions of the basis functions (Hilbert space), and partitioning of the total electron density in real space.^40^ To account for both families of charge models, we selected two charge models of each family for testing: (i) natural bond orbital analysis-derived charges (NBO), (ii) Mulliken charges (MUL), that are based on basis set partitioning; as well as (iii) electrostatic potential (ESP) and (iv) restrained electrostatic potential (RESP)-derived charges, that are based on fitting to the electrostatic potential in real space. Obtaining MUL charges is computationally inexpensive and simple, albeit highly dependent on the basis set.^41^ Nevertheless, they are commonly used for different applications, e.g., within extended semiempirical tight binding (GFN2-xTB).^42^ On the other hand, NBO charges are less dependent on the basis set and thus, generally more robust, while also taking into account polarizability.^43,44^ Partial charges obtained via the electrostatic potential (ESP and RESP) method are widely used within AMBER FFs.^38,45,46^ Here, RESP adds a restraint function during ESP fitting to decrease the charge strength that tends to be overestimated by ESP alone.^46^ To emphasize the importance of charge assignment, Liu et al. recently benchmarked five different partial charge fitting models for metal–organic frameworks (MOFs),^47^ underscoring the need to evaluate charge models based on their performance in fixed-point charge simulations relevant to practical applications.

First, different subgroups of atoms of the two differently *endo*-functionalized MCgs (*endo*-**C** and *endo*-**N**) were identified for the grouping of symmetry equivalent atoms, to assign averaged charges to equivalent atoms. For automation of the workflow, *TUCAN* analysis^48^ was performed for both MCg systems. The resulting atom grouping is almost identical, with the only difference coming from the different *endo*-atoms (−CH and −N), resulting in a different number of atom groups: 19 groups for *endo*-**C** versus 18 groups for *endo*-**N**. The results of the grouping are shown in Figure S1A,B.

In the case of *endo*-**C**, MUL charges produce the most polarized partial charges, with the *sp*-carbon atoms showing the most negative one (Figure S2A–C). Fluctuations of carbon atom partial charges ranging from −0.6 up to 0.4 can be seen within MUL charges, while NBO, RESP, and ESP carbon charges are in closer accordance with each other, as well as exhibiting a generally lower polarization between different carbons. Larger discrepancies between the charge models are visible for the metal-coordinating nitrogen atoms, as well as for the Pd-atoms themselves, i.e., for stronger polarized atoms. These trends were very similar for the *endo*-**N** cage, showing similar discrepancies in the same key atoms, but also slightly more polarized charges for the ESP charge model. The main discrepancy between the charge distribution of the two cage analogs arises from the different electronic properties, determined by the different *endo*-functionalization (Figure S2A–C).

To assess how different partial charge distributions affect the MCg structures, MD simulations were conducted using each charge model integrated into the force field. After equilibration (Figure S3), MD simulations were run for 100 ns producing 4 replicas per setup. As the MCgs repeatedly adapted different conformations during the simulations, relaxed energy scans were performed in water and DMSO to study the energetic difference between their conformers and their accessibility. The energy of the system was analyzed as a function of the distances *d1* and *d2* between the *endo*-facing atoms (N or C) (Figure S4). These could describe the three main minima in the potential energy surface corresponding to the three most recurrently observed conformations: namely, *closed*, *semiopen*, and *open* (Figures 3 and S8). These conformations naturally emerge from all simulations of the different combinations of the three force field generations, the four charge-fitting models, and the two solvents.

**Figure 3:**
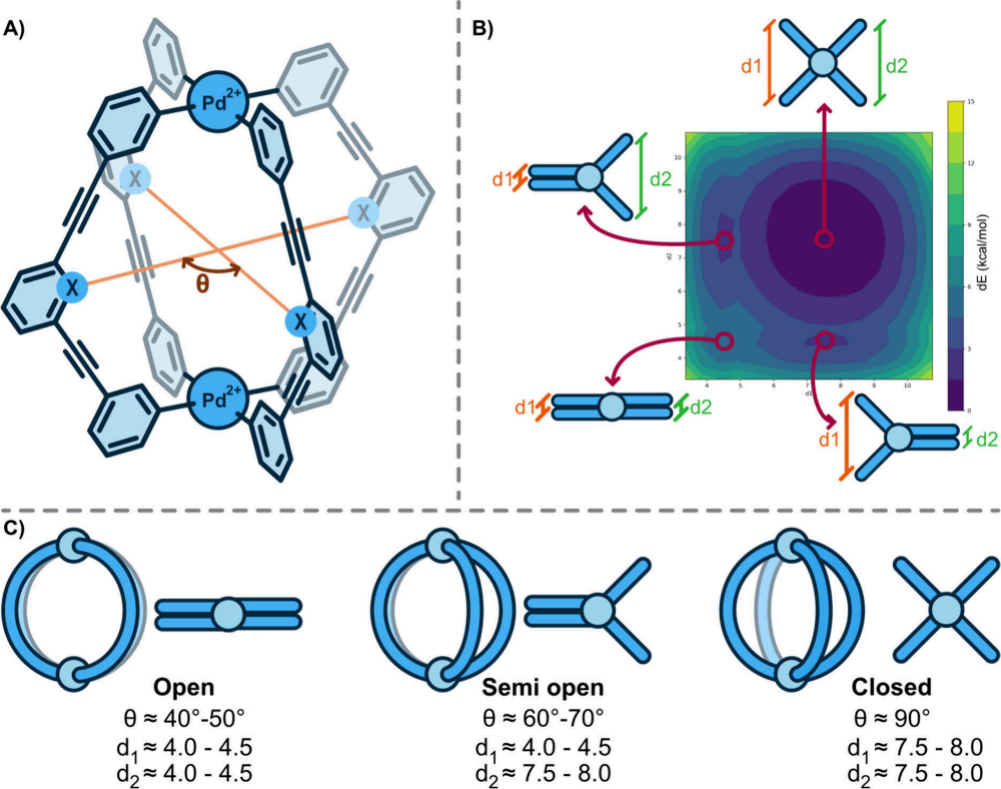
(A) Schematic representation of the selected [Pd_2_L_4_]^4+^ MCgs, and definition of angle θ. X = N or C. (B) DFT single points of *endo*-**C** structures obtained from relaxed xTB geometry scans in DMSO, showing the stability of the observed conformers corresponding to the distance (d1, d2) of adjacent ligands. Results for *endo*-**C** scans in water, and *endo-* **N** scans in DMSO and water can be found in the SI (Figures S5–S7). (C) Graphical description of the criteria used for the assignment of the three conformational states (from left to right: *open*, *semiopen*, and *closed*) as a function of the angle θ, observed during the MD simulations of both MCgs.

The three states were classified as follows:

1. *Open conformation*: π-stacking of two pairs of aromatic rings of neighboring ligands with an angle of θ ≈ 40–50°, resulting in the cage losing the cavity and forming a ring-shaped conformation. This occurs by rotation of the metal-coordinating pyridinyl unit while maintaining quadratic planar coordination to the Pd-atom, facilitating the stacking of the central aromatic rings of the respective ligands.
2. *Semiopen conformation*: lowest symmetry conformation featuring two ligands π-stacking on top of each other with an angle of θ ≈ 60–70°, and the other two ligands maintaining their original, not interacting, orientation.
3. *Closed conformation*: high symmetric cage conformation with an angle of θ ≈ 90° between the central aromatic rings of the ligands and a “closed” cage cavity.

The *open* conformation is the least stable one (dE_open‑closed_ = 4.85 kcal/mol) while the *closed* state is the most stable. The *semiopen* state represents an intermediate stability conformation (with dE_semiopen‑closed_ = 2.76 kcal/mol) between the *open* and *closed* states. Based on the calculations performed for the solvated model, we have identified a stable parametrization for AMBER FFs with the 2nd and 3rd generations. Evidently, the parameters of the 1st generation force field were not sufficiently optimized to adequately model the MCgs dynamics. However, for comparative purposes, and to highlight how changes in the force field impact subsequent results, it was still included in the discussion. While the 2nd generation force field already exhibits an improved cage representation, *open* and *semiopen* conformations were still observed to form repeatedly during MD simulations. In an endeavor to suppress the occurrence of these states, metal improper dihedrals were defined in the 3rd generation force field topology. Nevertheless, the rate of appearance of the *open* and *semiopen* states was similar to the 2nd generation force field, so no clear difference was achieved. Still, the 3rd generation force field was considered the latest version with the most robust parameters for interaction studies.

The dynamic evolution of the ligands’ angles over the simulation time and densities of the three conformational states were traced (Figure 4 and Figures S9–S11), showing that the conformational behavior of the *endo*-**C** and *endo*-**N** cages is strongly influenced by the choice of partial charges, solvent, and force field generation. Furthermore, population analysis displaying the residence time of the cages in the three different conformations was performed by integrating the state densities, in order to assess the likelihood of the occurrence of each state during the atomistic simulations (Figure 5).

**Figure 4:**
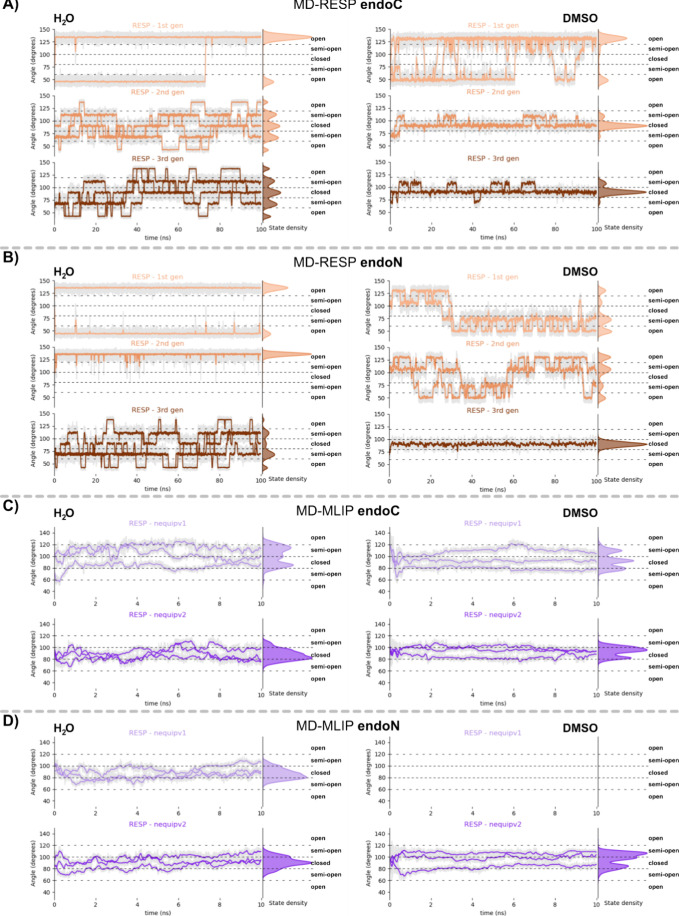
Comparison of the conformational changes observed over time during MD as a function of the angle between two adjacent organic ligands, and state densities of the *endo*-**C** (A, C) and *endo*-**N** (B, D) MCgs using classical FF, or NequIP *v1* and *v2* MD simulations. Analyses were performed for the three generations of force fields fitted with RESP partial charges for both solvents water (left) and DMSO (right), respectively. For each force field generation, four replicas of MD simulations were conducted, all contributing to the displayed distributions. The conformational changes during classical MD for both MCgs with the other charge models (MUL, NBO, ESP) are shown in the SI (Figures S9–S11). For each version of the MLIP, three replicas of MD simulations were conducted. Due to the instability of the MLIP *v1* for DMSO-solvated systems, they are omitted in the graph.

**Figure 5:**
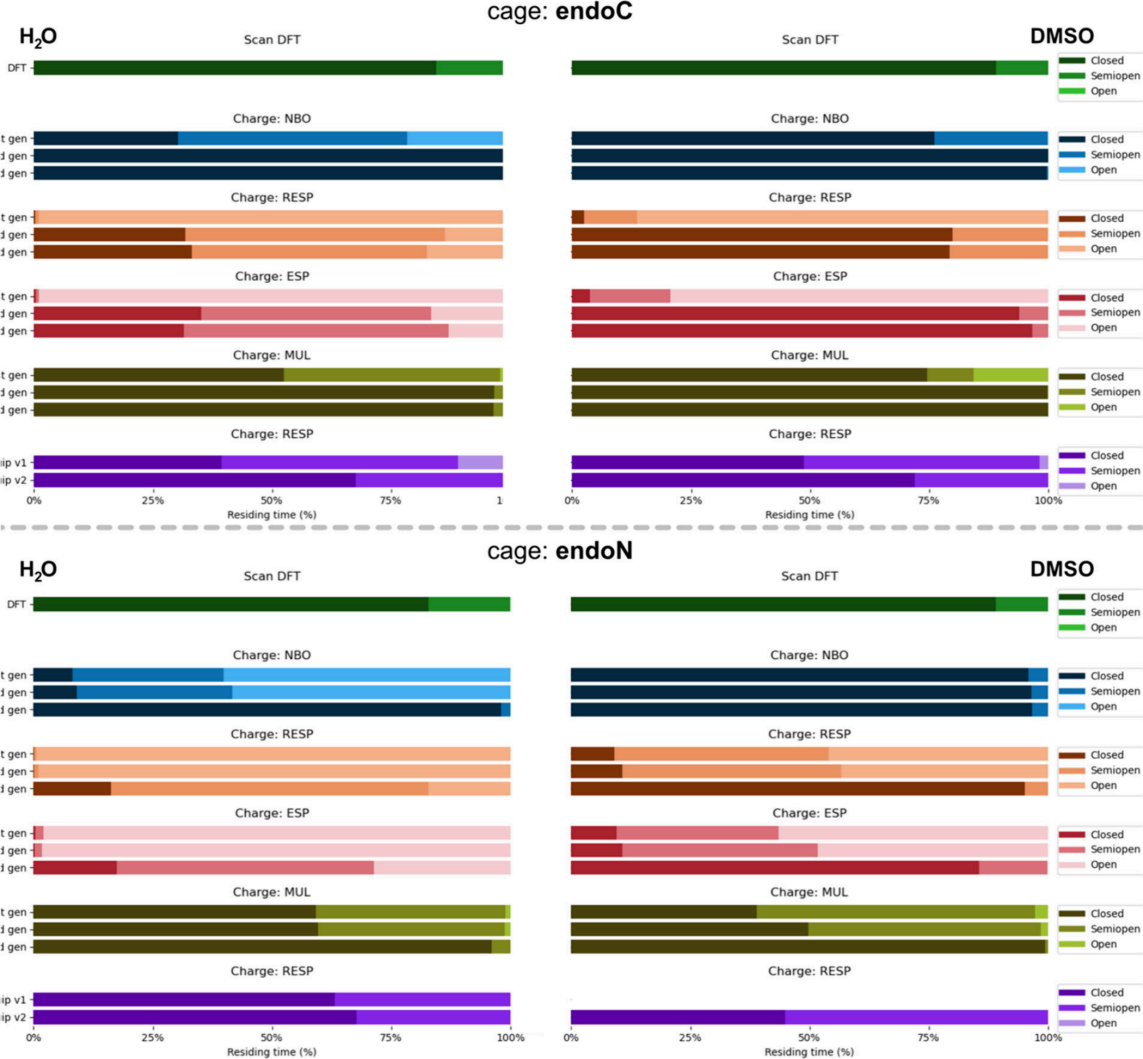
Comparison of population density of the three states *closed* (dark color), *semiopen* (medium color), and *open* (light color) during MD for DFT scans, all three generations of FFs, and NequIP MLIP *v1* and *v2*. Comparison for the *endo*-**C** (top) and for the *endo*-**N** (bottom) MCgs. Population analysis was performed for the MD runs in water (left) and DMSO (right) and for all of the four charge fitting models used within the FF generations and the MLIP.

Overall, water enhances conformational switching due to solvophobic effects and π-stacking, whereas DMSO promotes rigidity, stabilizing conformations via dipole–dipole interactions. As observed during the simulations (Figure 4A,B), conformational switching from *closed* to *semiopen* state occurs in DMSO for both MCg analogs. Due to the large energy penalty of folding into the *open* state, the latter is not observed during simulations in DMSO for the latest version of classical FFs. In the direct comparison of *endo*-**N** and *endo*-**C** cages in water, especially with respect to the more flexible ESP and RESP charge models, the *endo*-**C** system is less prone to adopt *semiopen* or *open* conformations, compared to *endo*-**N**. This can be rationalized by the more hydrophobic character of the fully formed *endo*-**C** cavity (Figure S12A), favoring a *closed* conformation. Conversely, *endo*-**N** exhibits a hydrophilic cavity (Figure S12B), allowing for solvent–solute hydrogen bonds, which induce ligand flexibility, as motion is crucial for energetically efficient bonding, but can at the same time affect the stability of encapsulation of a certain cargo.

For *endo*-**C**, ESP and RESP charges in water favor flexible, *semiopen*/*open* states; while DMSO stabilizes the *closed* cage form for the 2nd and 3rd generations FF, with the cage adopting the *semiopen* state only 3–6% (ESP) to 20% (RESP) of the time, respectively (Figures S9A and 5). NBO and Mulliken charges lead to a predominantly *closed* conformation in both solvents. A similar situation could be described for the *endo*-**N** cage, whereby NBO and MUL partial charges also led the MCg to exclusively adopt the *closed* conformation in both solvents (Figures 5 and S10–S11). In contrast, even when using the third-generation force field, ESP and RESP partial charges exhibit relatively short residence times in the *closed* state when solvated in water, while the *open* state is detectable for ∼29% (using ESP) and ∼17% (using RESP) of the time. In DMSO, the time spent by *endo*-**N** in the *closed* state is significantly higher (∼86% using ESP and ∼95% using RESP partial charges) and could be retained for most of the simulation time using the third-generation FF, while the first and second-generation FFs produce predominantly *semiopen* and *open* conformations. In general, independent of the cage analog, the third-generation FF better captures solvent-dependent dynamics and state transitions.

To enable a more direct comparison of cage flexibility between MD simulations, Boltzmann populations derived from DFT scans were included in Figure 5. Populations of the 225 structures obtained from the scans were computed (see Supporting Information), classified by conformation (*open*, *semiopen*, *closed*), and summed to provide a comprehensive reference data set. Since the two methods to calculate the population are substantially different (MD residence time vs static Boltzmann population with implicit solvent), the results do not match perfectly, and comparisons should be made cautiously. However, due to the cost of performing a DFT-based MD, a closer approximation is not feasible. The presence of *open* conformations in classical MD can be reasoned by an overestimation of energies coming from certain FF and charge combinations, showing and quantifying an inaccurate description of the dynamic behavior by the classical methods.

To further probe these conformational dynamics and evaluate the balance between accuracy and computational efficiency, we also employed MLIPs. Two NequIP-based models (*v1* and *v2*) were trained with *v2* featuring a ∼ 1.5 times larger model due to one additional message-passing layer and a higher spherical order of equivariant features for improved force prediction (see SI, Figure S13). As with the classical FF, the MLIPs can be used to calculate energy and forces given an atomistic structure, enabling a direct comparison between classical and machine learning-assisted approaches.

Visibly, in Figure 4C,D and Figure 5, the MD simulations of the MLIP in water render conformations only in the *closed* and *semiopen* state for both MCg analogs. While the *open* conformation is observed very shortly during simulation with MLIP *v1* (Figure 5), during simulations with the more robust MLIP *v2*, the *open* state cannot be detected. Additionally, the conformational populations feature smoother transitions between each conformation. This is likely due to the fact that classical MD parameters strive to maintain stability of the system throughout the simulations, which is achieved by introducing adequate force constants modeling harmonic potentials, and thus, leading to more rigid conformations and, consequently, more abrupt switching between states. In contrast, MLIP-based simulations enable anharmonic modeling with a more flexible dynamic behavior, thereby avoiding the previously observed sudden changes between states.

For MLIP v2 in DMSO, both cage analogs behave very similarly to the simulations in water, again featuring smooth transitions between the *closed* and *semiopen* states. It has to be noted, that for the MLIP runs, due to computational cost, three replicas of only 10 ns simulations were produced; while for classical FF three replicas of 100 ns simulations were obtained. In addition, the simulations of MLIP *v1* in DMSO rendered instabilities and were therefore, omitted from further analysis. Interestingly, the open conformations are almost absent in the MLIP simulations, most likely due to their higher energy (as shown by the DFT results and single point energies in Figure 6).

**Figure 6:**
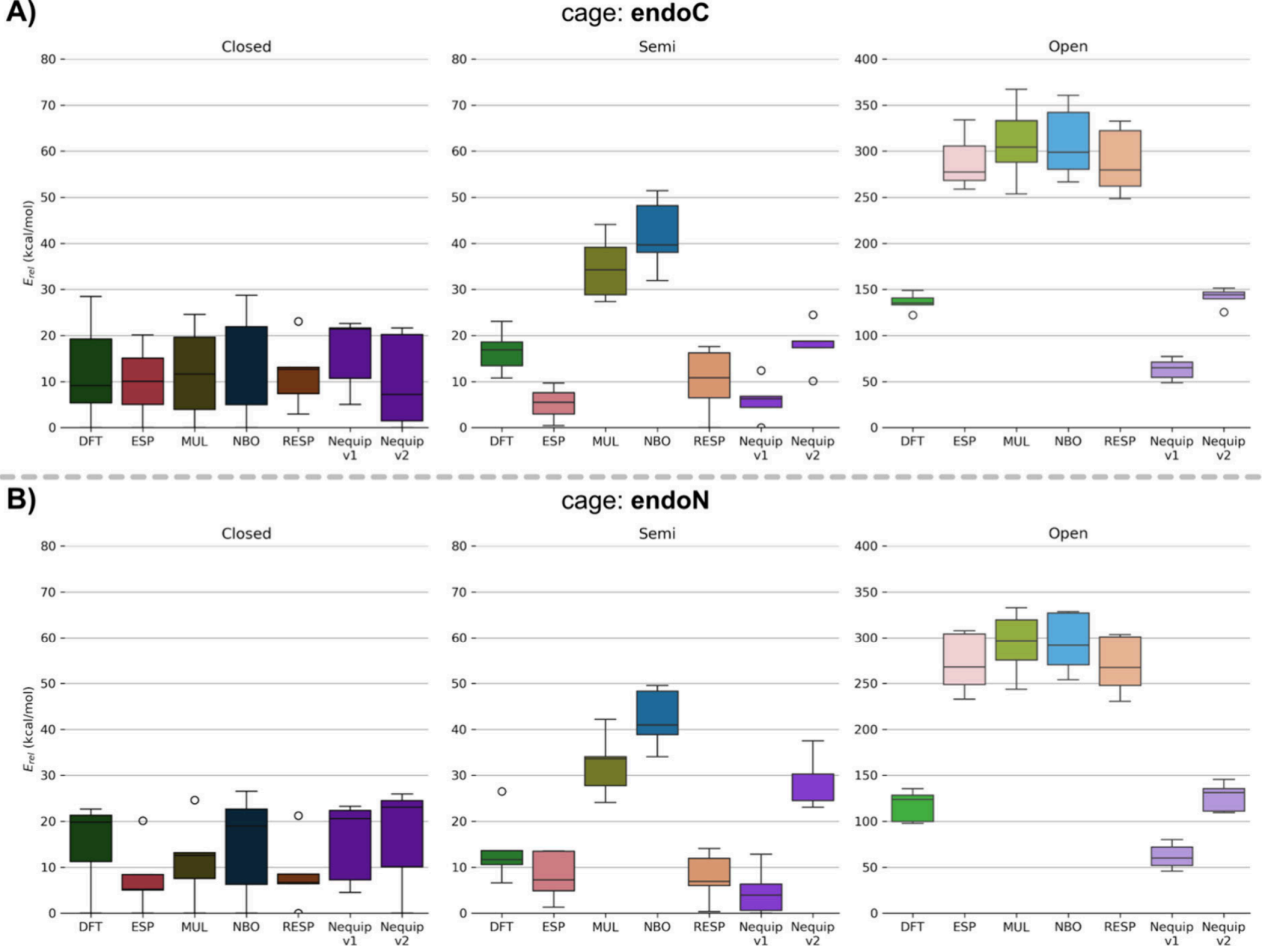
(A) Box plots of energy difference relative to the lowest energy conformer for all four partial charge models (ESP, MUL, NBO and RESP) as well as for relative energies calculated in DFT and with NequIP MLIP *v1* and *v2* for the *endo*-**C** analog. (B) Box plots of energy difference relative to the lowest energy conformer for all four partial charge models (ESP, MUL, NBO and RESP) as well as for relative energies calculated in DFT and with NequIP MLIP *v1* and *v2* for the *endo*-**N** analog. Shown are the energies in the closed state relative to the lowest energy conformer (left), as well as for *semiopen* (middle) and *open* states (right), respectively.

Finally, to assess the accuracy of both classical FFs and MLIPs in describing the MCg analogs, single-point energy calculations were carried out on five representative frames from each conformation (*closed*, *semiopen*, and *open*, Figure 6) by DFT, molecular mechanics with third-generation FFs, and the two MLIP models (*v1* and *v2*), respectively. For consistency, all single-point energy evaluations were conducted in the gas phase.

In this comparison, two factors must be considered relative to the DFT reference to evaluate the accuracy of the method when evaluating the energy of the system: (i) the agreement of the median values and (ii) the similarity of the distributions (quartile range, whiskers, and outliers).

For the *closed* state of *endo*-**C**, all charge models, as well as DFT and the MLIP models are in close accordance with each other, exhibiting relative potential energies of *E*_rel_ < 30 kcal/mol (Figure 5, top panel). When looking at the *semiopen* conformation, RESP and ESP charge models show a relative energy difference of *E*_rel_ < 20 kcal/mol, which is in the same range as the relative energies of DFT (*E*_rel_ = 10–23 kcal/mol) and NequIP MLIPs, *v1* (*E*_rel_ = <12 kcal/mol) and *v2* (*E*_rel_ = 10–25 kcal/mol), while NBO and MUL charges seemingly lead to an overestimation of the potential energy with relative energies in the range of *E*_rel_ = 28–51 kcal/mol. Relative Energies for the *open* conformation described by the classical FFs are in a much broader range of *E*_rel_ = 248–380 kcal/mol, compared to DFT and the two MLIP versions (Figure 5, top panel). The wide energy ranges observed for the *closed* and *open* states reflect a greater variability and uncertainty in the energies derived from MD simulations, whereas the narrower distributions for the *semiopen* state suggest more consistent energy values across all five replicas. Nonetheless, both DFT and the NequIP MLIP models (*v1* and *v2*) display tightly clustered energy values within each conformational state, indicating higher internal consistency.

The DFT calculated energies for the *open* states provide a narrower distribution with *E*_rel_ = 122–149 kcal/mol. NequIP MLIP *v1* energies are lower with *E*_rel_ = 49–77 kcal/mol. Notably, NequIP MLIP *v2* shows the best performance by almost identical reproduction of the DFT energies (*E*_rel_ = 125–151 kcal/mol). Summarizing, the distribution and mean in the *closed* state of the classical FF-based methods and the MLIP are in close accordance with the DFT values. In *semiopen* and *open* states, the discrepancy in mean energies and overall distribution of the classical FF-based methods are much higher and broader than the DFT values, with a difference of ca. 100 kcal/mol in the *open* state. Nevertheless, despite yielding significantly higher absolute energies than DFT, the classical FFs using ESP and RESP-derived charges show smaller energy differences between conformers, making their performance more comparable to DFT than that of MUL and NBO charge models. Overall, the energy values and distributions obtained with MLIP *v2* align most closely with the DFT results.

In the case of the *endo*-**N** analog in the *closed* state, all conformers lie in close proximity with *E*_rel_ < 30 kcal/mol and thus, exhibit only small deviations to the reference over all tested charge models, as well as DFT and NequIP MLIP versions. As for the *semiopen* state, the potential energies *E*_rel_ are between 28 and 50 kcal/mol for NBO and MUL charges, while for RESP and ESP charges, *E*_rel_ is only up to 14 kcal/mol. The same is observed in the case of the NequIP MLIP *v1* with *E*_rel_ < 13 kcal/mol in the *semiopen* state, while NequIP MLIP *v2* provides energies of *E*_rel_ = 23–37 kcal/mol. This is in close accordance with the DFT values (*E*_rel_ = 6–27 kcal/mol). In parallel, the energies obtained for all charge models tested for the fully *open* conformation spread relatively broad in the range of *E*_rel_ = 230–333 kcal/mol. This suggests the existence of multiple *open* conformations that differ vastly in their potential energy. A similar trend can be observed for the DFT calculations with the energies *E*_rel_ being around 98–136 kcal/mol in the *open* conformation. In contrast, NequIP MLIP *v1* exhibits an energy difference of *E*_rel_ = 46–80 kcal/mol, following the trend already seen in Figure 6A, where lower energy values are obtained by DFT. Further, NequIP MLIP *v2* matches more closely the DFT values with *E*_rel_ = 110–146 kcal/mol. Thus, ESP and RESP-derived partial charges within the classical force field lead to energies closer to the DFT quantum energies than MUL and NBO, albeit all still being significantly larger than DFT overall. In summary, the classical FF models of *endo*-**N** provide, in line with what shown for *endo*-**C**, ca. 100 kcal/mol higher energy values for the *open* state than DFT. NequIP MLIP-based energies show a better accordance with DFT results compared to classical force field methods, albeit not as close as in the case of the *endo*-**C** cage analog for the *semiopen* state (Figure 5). Across all simulation conditions, the *open* conformations consistently show the highest energies among all states, which explains their absence in MD simulations using the trained MLIP. While MLIP achieves energy predictions with accuracy close to that of DFT and surpasses classical FFs in this regard, it comes at the cost of significantly higher computational demand.

## Conclusion

In this work, we developed and applied a multilevel computational workflow to study the structural dynamics and conformational preferences of two [Pd_2_L_4_]^4+^ MCgs, *endo*-**C** and *endo*-**N**, with a focus on their potential use in guest encapsulation and drug delivery applications. Through extensive MD simulations, we systematically benchmarked four widely used fixed-point partial charge models - NBO, Mulliken (MUL), ESP, and RESP - using classical FFs and compared their performance against MD simulations using two versions of the NequIP Machine Learning Interatomic Potential (MLIP). All atomistic simulations were carried out in both water and DMSO to assess the influence of the solvent environment on conformational behavior. The solvent’s impact on the conformational stability of the MCgs also affects their ability to encapsulate and retain small molecules within the cavity, which is crucial for potential drug delivery applications.^49^

From the MD simulations, MCgs switching between conformations emerges, comparable to the “breathing” behavior of MOFs observed while external stimuli are applied, such as exposure to different solvents.^50^ Our analysis focused on quantifying the populations of the *closed*, *semiopen*, and *open* conformations of the cages under various conditions. Among the classical approaches, RESP and ESP charges showed the best agreement with DFT reference data in terms of energetic ordering and conformational sampling, particularly in capturing solvent-induced transitions. Furthermore, MLIP-based simulations demonstrated excellent agreement with DFT energetics, especially with the improved *v2* model, and largely reproduced the most stable *closed* state conformations. For the *closed* conformations, classical FF methods perform comparably well with MLIP methods, i.e. ESP and RESP methods for *endo*-**N** in the *semiopen* state. As the *closed* and *semiopen* states are most frequently observed, we can show good agreement between the simulations run through classical FF and the DFT references. However, MLIPs show a smoother transitioning between the states, as opposed to the abrupt conformational changes observed for the classical MD, especially for the best performing cases, ESP and RESP. The improved accuracy of MLIPs-based simulations comes at the expense of higher computational cost compared to classical FFs, though it still remains lower than that of *ab initio* molecular dynamics simulations. While classical MD simulations reach a maximum performance of 113.9 ns/day, the MLIP-based simulations only reach a maximum of 1.8 ns/day. On average, classical methods allow for a simulation speed of 80 ns/day, which exceeds the average speed for the MLIPs (1.2 ns/day) by almost 2 orders of magnitude. Additional details on the comparison of computational cost can be found in the SI (Figure S14).

In summary, our results show that *open* conformations are unlikely to be observed in DMSO, but appear in water simulations. DMSO stabilizes the *closed* state through dipole–dipole interactions, preventing full opening and aligning with MLIP predictions. In contrast, water promotes cage flexibility via hydrophobic effects and π-stacking, possibly favoring guest encapsulation. The *endo-* **C** cage remains more rigid due to its hydrophobic cavity, while the hydrophilic *endo*-**N** cage shows greater flexibility from solvent–solute hydrogen bonding. The latter feature, may, however, partly account for the experimentally observed scarce retention of encapsulated cargoes (cisplatin) in *endo*-**N** type Pd_2_L_4_ MCgs.^9^ This finding is important because, until now, the preferential encapsulation of cisplatin in a Pd­(II) cage’s hydrophobic cavity has mostly been attributed to the tendency of “hydrogen-bond frustrated” water molecules (or other polar solvents) to reside outside the cavity.^15^ Our results instead point toward a more complex interplay of solvent effects and void’s polarity on both the MCg structural dynamics and enthalpy-driven guest binding, ultimately affecting the host–guest capabilities.

Overall, our study highlights the critical importance of charge model selection in classical MD for metal–organic assemblies, as well as the promising potential of MLIP methods for achieving DFT-level accuracy in dynamic simulations. These initial insights pave the way for further and currently ongoing studies on the host–guest interactions of small chemotherapeutic drugs encapsulated in the MCgs’ cavities for targeted drug delivery. The presented workflow provides a transferable framework for the computational screening and design of supramolecular systems in solution, bridging the gap between quantum accuracy and the time- and length-scale requirements of simulations relevant to functional applications, such as molecular encapsulation and targeted delivery.

## Supplementary Material



## Notes

Additional information on computational details of the methods applied, including figures for population analysis and dynamic evolution of angles over simulation time for all charge and force field models, as well as figures describing system setups and information for the Zenodo repository for this publication (PDF)
