# Supplementary material for: Computational Workflow to Unravel the Structural Dynamics of Supramolecular Metallacages in Solution
Source: J Chem Theory Comput. 2025 Nov 20;21(23):12278–88. doi: 10.1021/acs.jctc.5c01465 (PMC12874367; doi:10.1021/acs.jctc.5c01465)
Supplement: Supplementary file 1 [file ct5c01465_si_001.pdf]

SUPPORTING INFORMATION

A Computational Workflow to Unravel the Structural Dynamics of Supramolecular Metallacages in Solution

Julia A. Stebani†[1], Íñigo Iribarren Aguirre†[2], Gohar A. Siddiqui[2], Darren Wragg[1], Alessio Gagliardi*[2,3] and Angela Casini*[1,3]

[1] Medicinal and Bioinorganic Chemistry, Department of Chemistry, School of Natural Sciences, Technical University of Munich, Lichtenbergstr. 4, 85748 Garching b. München, Germany.

[2] Simulation of Nanosystems for Energy Conversion, Department of Electrical Engineering & Atomistic Modeling Center (AMC), School of Computation, Information and Technology, Technical University of Munich, Hans-Piloty-Str. 1, 85748 Garching b. München, Germany.

[3] Munich Data Science Institute (MDSI), Technical University of Munich, Walther-Von-Dyck Str. 10, 85748 Garching b. München, Germany.

Computational Details

Metallacage construction

All the metallacages were constructed using the M_2L_4 lantern-shaped template available in the supramolecular toolkit (STK) python library[1] in combination with the MCHammer[2] optimizer. The script used can be found in the supporting information (Script S1 on the zenodo repository).

Density Functional Theory calculations

Relaxed geometric scans on the metallacage *endo*-**C** and *endo*-**N** were performed to ensure the obtention of the most stable conformation for each cage. These scans were performed in *ORCA*[3] using semiempirical tight-binding method (GFN2-xtb)[4]. Implicit solvents water and dimethyl sulfoxide (DMSO) were used within the ALPB[5] solvent model for xTB. For the scans, the length of the two distance vectors connecting carbon atoms C2 and C4, as well as carbon atoms C6 and C86 was increased in increments of ca. 0.5 Å starting from an initial distance of 3.36 Å (C2-C4) and 3.39 Å (C6-C86). With the final distance being 10.75 Å for both distance vectors, this results in 15 steps for the complete scan (for detailed descriptions of the atom positions scanned, see Figure S4. The scan results are shown in Figures S5-S7.

Single-point calculations were performed on each of the structures at the PBE[6,7]+D3Zero[8]/def2-SVP[9] level using SMD[10] implicit solvent model for water and DMSO. The three minima identified were re-optimised at the same level of theory in order to obtain the relaxed structures of the absolute and relative minima.

The population of the individual structures extracted from the scans is calculated following the equation:

$$p_i = \frac{e^{(-\Delta E_i/RT)}}{\sum_i^N e^{(-\Delta E_i/RT)}}$$

Single-point calculations for energy comparison in gas phase were performed at the same level of theory.

FF parametrization

Three generations of the force field were created, named *first*, *second* and *third generation*.

- *1st generation*: The first generation force field was established by the Seminario method[11] within the *automated force field topology builder* (ATB) software.[12] With this, AMBER force field

parameters were obtained for the organic ligands, for either *endo*-**C** or *endo*-**N** analogs. The parameterization of the Pd^{2+} metal site and Lennard-Jones parameters was done in accordance with results published previously.[13]

- *2nd generation*: Due to poor initial cage stability, parameters were adapted according to more recent publications.[14,15] Also, symmetry-equivalent atoms were determined using TUCAN[16] Analysis and subsequently given the same bonded and non-bonded parameters.
- *3rd generation*: Improper dihedrals having the Pd^{2+} metal as the central atom were added to the force field to assess a possible improvement of the cage cavity stability.

All the FF parameters can be found in the Files S2 - FF parameters folder in Zenodo.

Atomic charges and identification of equivalent atoms

Atomic partial charges derived from natural bond orbital (NBO) analysis were obtained using the *Gaussian/NBO7*[17–19] software (B3LYP[20–23]/6-31G(d,p)[24]/LAN-L2DZ[25–28]). Atomic partial charges derived from Mulliken population analysis[29,30] (MUL) were obtained using the CP2K[31] software (PBE0[32]-D3/6-31(d,p)/LAN-L2DZ). Atomic partial charges derived from the electrostatic potential (ESP)[33] were obtained from the *Jaguar* software[34] from the *Schrödinger* suite (PBE0-D3/6-31(d,p)/LAN-L2DZ and B3LYP-D3/6-31(d,p)/LAN-L2DZ). Atomic partial charges from restrained electrostatic potential (RESP)[35] were taken from previous reports for the same structure[15], and generated within *metallicious*[14] using *psiRESP*[36]. Charges were then averaged over symmetry-equivalent atoms using *TUCAN*[16] analysis for atom grouping.

The raw and averaged atomic charges can be found in the Files S3 - Atomic charges folder in Zenodo.

FF creation

The atomic partial charges for the two metallacage analogs were calculated using different charge fitting methods (Mulliken (MUL), electrostatic potential (ESP), restrained electrostatic potential (RESP), and natural bond orbital (NBO)) and their performance subsequently compared by performing MD simulations. To this aim, the force field of each generation was modified by substitution of the partial charges assigned to each atom in the topology file generated by GROMACS *pdb2gmx*. The workflow depicted in Figure 2 (Main Text) was therefore applied to every generation of the FF and charge fitting model, resulting in 12 unique FF that were used for the simulations.

Classical Molecular Dynamics

Starting conformations for the Molecular Dynamics (MD) simulations were obtained from optimized structures of both metallacages, *endo*-**C** and *endo*-**N** with tetraflouroborate (BF_4^-) counter anions to counterbalance the positive charge, in either DMSO or water as solvents. The simulations were performed in GROMACS[37] (version 2022.3) inside a 40 x 40 x 40 Å box with periodic boundary conditions (PBC). During equilibration, the systems were solvated either with ca. 2100 water molecules, using the TIP3P water model, or with DMSO (ca. 460 molecules). The 12 unique, adapted, Amber99sb-ILDN-slipid forcefields[38] were used. Long-range electrostatic interactions were calculated using the Particle-mesh Ewald (PME) method. The Verlet cut-off scheme was used for short-range electrostatic and van der Waals interactions with a cut-off distance of 8 Å. LINCS[39] algorithm was used for constraining bond lengths. For temperature and pressure coupling, the Nosé-Hoover thermostat[40] (τ = 0.5 ps at 300 K) and the Parrinello-Rahman barostat[41] (τ = 0.2 ps at 1 bar) were applied. The system was equilibrated in the NPT ensemble for 100 ps (details on the equilibration are shown in Figure S3). Production runs were run for 100 ns each with a timestep of 0.5 fs. For each unique force field 4 MD replicas were simulated, resulting in 48 MD simulations overall. The Molecular Dynamics Parameter (mdp) files for each step can be found in the Files S4 - MD setup files, and the MD topology and initial structures in the Files S5 - MD input files folder in the Zenodo repository.

Single-Point Energy with Molecular Mechanics

Single-point energy calculations for each of the four charge models within the *3rd generation* force field were performed using the GROMACS command *gmx energy*.

Machine Learning

As the workflow for the Machine Learning interatomic potential (MLIP) was very similar to the classical approach, only the steps that differ from the previously described classical workflow are described in detail below. The MLIP was trained using an iterative approach, which couples the calculation of energies and forces using DFT, the training of ML models using *pytorch* library[42] and using the model in MD simulations.

DFT calculations for MLIP training

The DFT simulations were performed using the CP2K[31] package. GTH pseudopotentials were used with TZVP-MOLOPT basis sets. The PBE exchange-correlation functional was used with D3 dispersion correction. Single-point calculations of the isolated cage were performed using the Gaussian Plane Wave (GPW) method[43] with a convergence threshold of 10E-6 for the self-consistent field.

MLIP training

The machine learning model *NequIP*[44] was used for the MLIP. The model has an E(3)-equivariant architecture, making it highly suitable for the prediction of forces for MD. We used a neighboring cutoff of 5 Å. For node features, we used a feature vector of length 32. The spherical harmonics part of the basis functions retained all terms up to the angular rank of 2 (l=2) and only the term with odd parity. The radial part of the basis function consisted of 8 Bessel basis functions with a polynomial envelope of order 6. We trained the bessel root during training. The radial basis was constructed with 2 layers each with 64 neurons. A total number of 4 interaction layers were used with an initial training rate of 0.005, which is reduced by a factor of 0.5 on plateau with patience of 50. A force/energy loss weighing of 99:1 was used, which was found to perform much better than equal weights. We used 10% of the available data for validation. For detailed information on the architecture of NequIP, please refer to the original source by *Batzner et al.*[44] A training batch size of 8 and validation batch size of 5 was used. An exponential moving average with a decay of 0.99 was used to update the network weights. The network is trained for a maximum of 5000 epochs, but we applied an early stop criterion on the learning rate of 1e-7, which stopped the training for all the models in the 1000-1500 epoch range.

The training was performed iteratively. For each iteration, a total of 10 new configurations were added to the training set of the MLIP, and it is trained from scratch, keeping all the hyperparameters the same. Initial structures for the training of MLIP were generated by taking the DFT-optimized structure and randomly perturbing each atomic coordinate with value between -0.08 and 0.08 Å. In the first iteration, energies and atomic forces for 10 such structures were obtained from the single-point DFT calculation. These were then used as dataset for the ML model training. The trained model was then used in the MD integrator to run MD simulations for 1 ps. The change in the structure during this MD run was analyzed. Different criteria were used to select one configuration from each of the 10 runs to be added to the training set of the ML model. For the first 10 iterations, the first frame that reached an

RMSD distance of 0.7 from the starting structure was selected. This is to develop a basic dataset that can train a model that manages to keep the bonded atoms together. For the subsequent iterations, always the last configuration at 1 ps is taken. Additionally, an external bias was applied to distances between the centers of the rings in the middle of the 4 arms of the cage to facilitate exploration of the *semi-open* and *closed*-cage configurations. Once all the MD simulations were completed, the next iteration started with single-point DFT calculations of the newly added structures. We kept track of the maximum RMSD of the newly added structures with the ones already present in the dataset and stopped the iterative procedure when it fell below a threshold. This resulted in a total of 22 iterations. The MLIP trained in the final iteration was used for further MD simulations.

For the simulations, two versions (*v1* and *v2*) of the MLIP were used that only differ in their setup of hyperparameters:

- *version 1: num_layers* = 3, *l_max* = 1, *num_features* = 32
- *version 2*: *num_layers* = 4, *l_max* = 2, *num_features* = 64

with *num_layers* being the number of interaction layers in the message passing step of the neural network, *l_max* the maximum order of the spherical harmonics basis term that was retained and *num_features* the length of the node feature vector. Therefore, the MLIP *v2* had double the length of the node features compared to *v1* (i.e. 64 instead of 32) and one additional message passing interaction layer. Furthermore, the network in *v2* retained basis set terms up to l=2 in the interaction steps, making it more efficient in retaining higher-order structural information.

MLIP stability criteria

The stability of the trained MLIP was evaluated based on a 1 ns run of cage in gas phase where the bond lengths of all the covalent interactions were recorded. If the bond lengths were in a tolerance range (+-5%) of the DFT optimized structure, the MLIP was used for the next steps.

MLIP Molecular Dynamics

MD simulations were performed using the LAMMPS package[45] in an NVT ensemble (Figure S13) with closed boundary conditions. A timestep of 0.5 fs was used. For each simulation, an energy minimization was performed for 1000 steps before the dynamics and 1000 more steps to raise the temperature. A Langevin thermostat[46] with a damping factor of 0.1 was used to keep the temperature at 300 K. The external bias during the MLIP training was applied using the OPES_METAD method of the PLUMED

plugin. A barrier height of 300 kJ/mol with a deposition rate of 5 fs was used. The final MLIP was directly used to obtain single point energies of isolated cage structures to compare with DFT and classical force field models.

The final MLIP was also used to perform longer MD simulations solvated in water. For this, a cubic box of side length of 33 Å with periodic boundary conditions in all directions was filled with 500 TIP3P water molecules and the cage at the center and 4 Cl- ions to neutralize the excess positive charge of the Pd atoms. The neighbor cut-off of 5 Å in the MLIP ensures avoidance of any self-interactions of the cage. All interatomic interactions of the cage atoms were taken into account by the MLIP. The atoms of the water molecules interacted by the classical TIP3P CHARMM model from *Jorgensen et al.*[47] The interaction between the cage and the water molecules are modelled with Coulomb and Leonard-Jones (LJ) potentials. The charges for the Coulomb interactions were taken the same as the ones calculated using the RESP method for the classical force field. The LJ parameters were taken from the AMBER-99 force field (ff99SB-ILDN)[38]. Three separate 10 ns MD runs for each of *endo*-**C** and *endo*-**N** were performed.

Simulation performance comparison

- Classical FF mean performance: 79.99 (ns/day)
- MLIP FF mean performance: 1.23 (ns/day)

- Classical FF fastest performance: 113.93 (ns/day) for 1st gen. RESP FF: *endo*-**C** in DMSO
- MLIP FF fastest performance: 1.76 (ns/day) for MLIP *v1*: *endo*-**C** in DMSO

- Classical FF slowest performance: 59.58 (ns/day) for 2nd gen. MUL FF: *endo*-**N** in water
- MLIP FF slowest performance: 0.51 (ns/day) for MLIP *v1*: *endo*-**N** in water

MLIP Single-Point Energy

Single-point energies for all conformers in the three states *closed*, *semi-open* and *open* were calculated from the trained MLIP energies and forces.

Figures

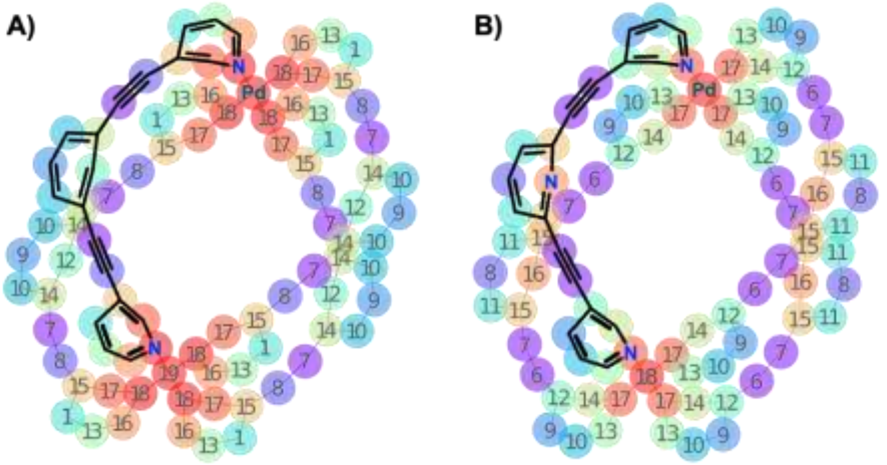

Figure S1. **A**) *TUCAN* Analysis of *endo*-**C** MCg analogue for atom grouping to derive equivalent atomic partial charges. Here, atom numbers 19 represent the Pd^{2+}-atoms coordinated to the four organic ligands. **B**) *TUCAN* Analysis of *endo*-**N** MCg for atom grouping to derive equivalent atomic partial charges. Equivalent atoms are displayed as similarly colored and with the same index. H atoms have been omitted for clarity.

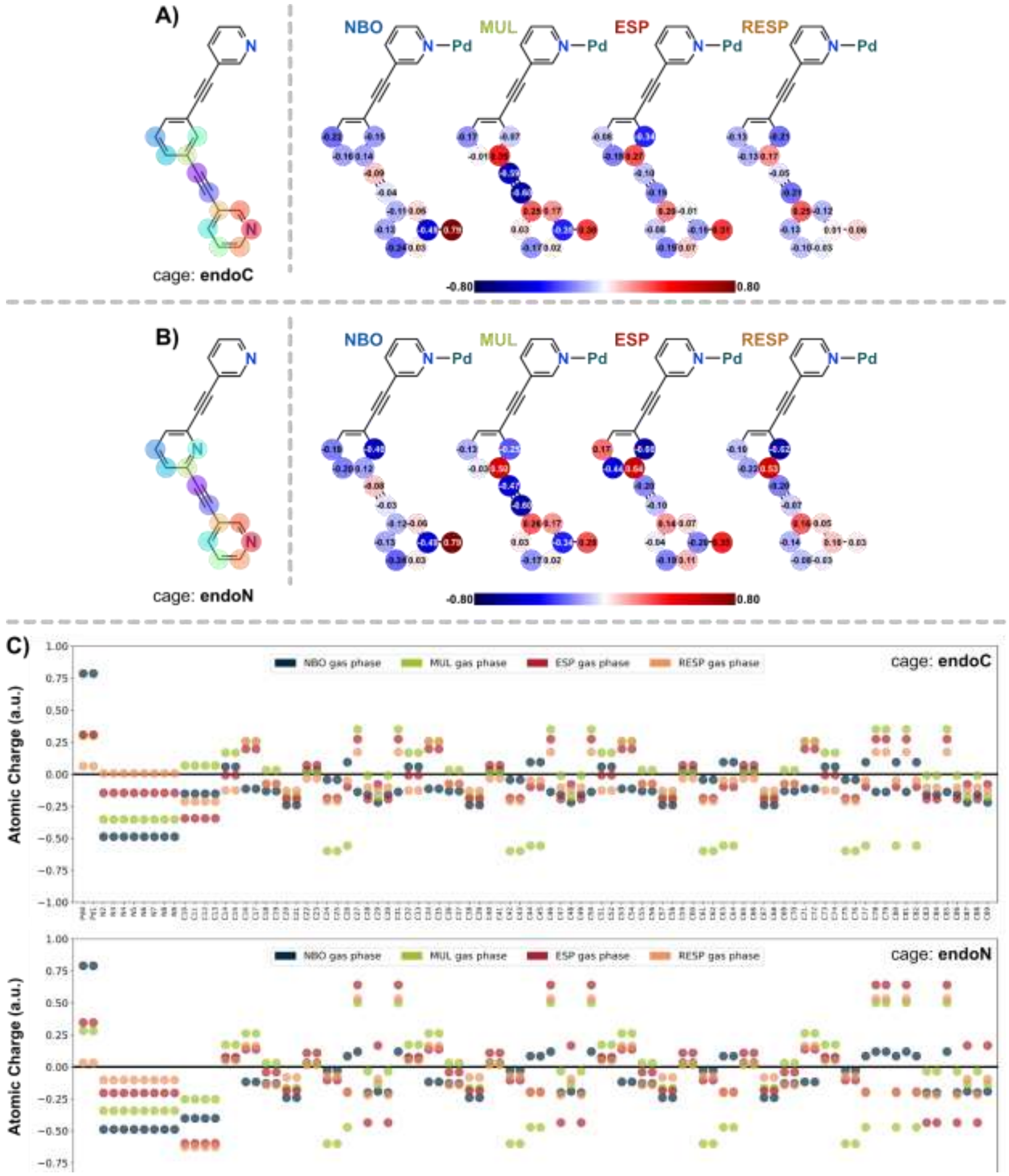

Figure S2. A) Distribution of partial charges for every atom in the *endo*-**C** cage analogue for the four charge-fitting models NBO (blue), Mulliken (green), ESP (red) and RESP (orange). Due to clarity, hydrogen atoms have been omitted. **B**) Distribution of partial charges for every heavy atom in the *endo*-**N** cage analogue for the four charge-fitting models NBO (blue), Mulliken (green), ESP (red) and RESP (orange). For clarity, hydrogen atoms have been omitted. **C**) Distribution of averaged partial charges on all the individual ligand atoms and the Pd^{2+}-atoms for both cage analogues *endo*-**C** (top) and *endo*-**N** (bottom) according to the four charge-fitting models NBO (blue), Mulliken (green), ESP (red) and RESP (orange).

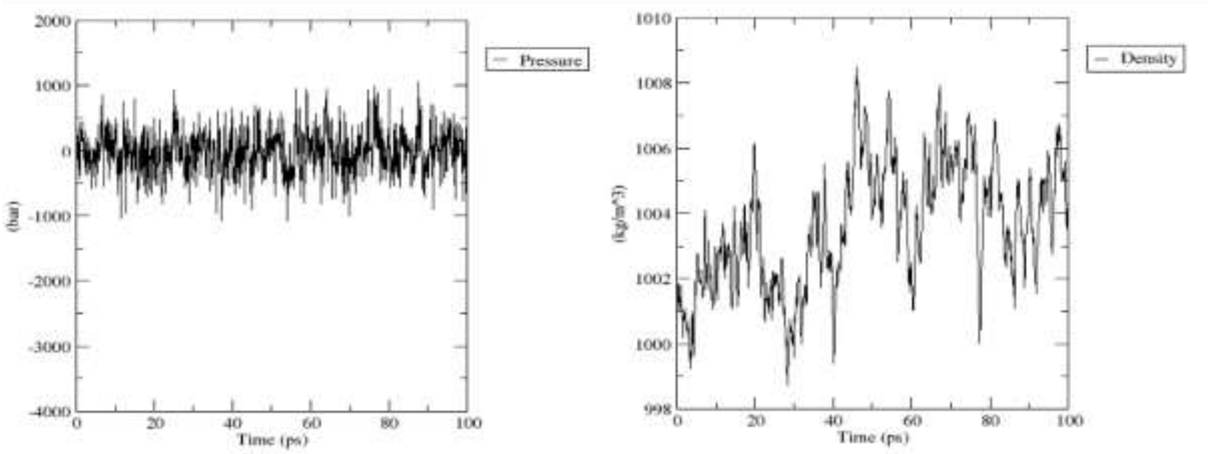

Figure S3. Equilibration of system pressure (left) and density (right) in GROMACS, prior to simulation run productions, of the *endo*-**C** MCg with NBO charges in water, using the third-generation classical force field. All other setups and simulations in DMSO reported in this paper were equilibrated in the same way.

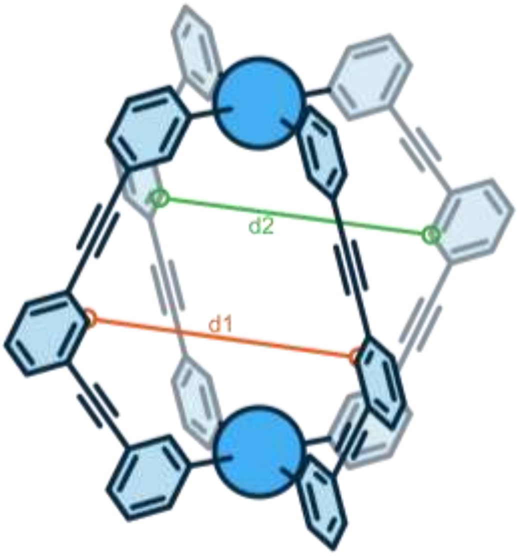

Figure S4. Schematic depiction of the distances *d1* and *d2* between the *endo*-facing atoms (N or C) varied during energy scans of both MCgs (*endo*-**N** and *endo*-**C**).

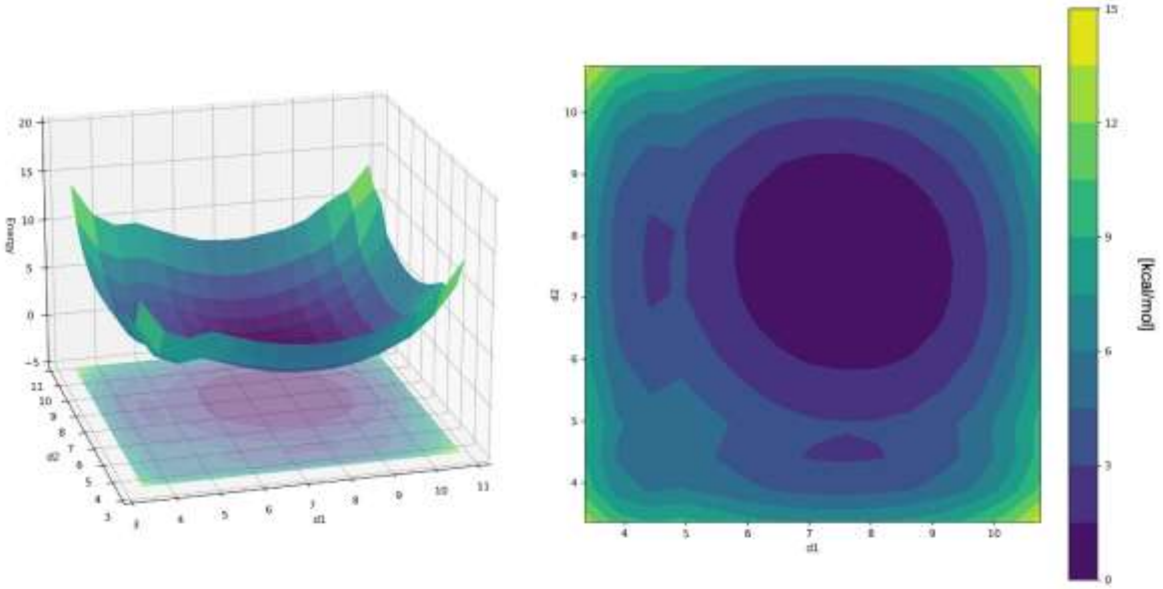

Figure S5. 3D plot (left) and heatmap (right) of DFT relaxed energy scans of *endo*-**C** conformers obtained from xTB showing the stability of the observed conformers corresponding to the distance (d1, d2) of adjacent ligands. DFT and xTB scans were performed in water.

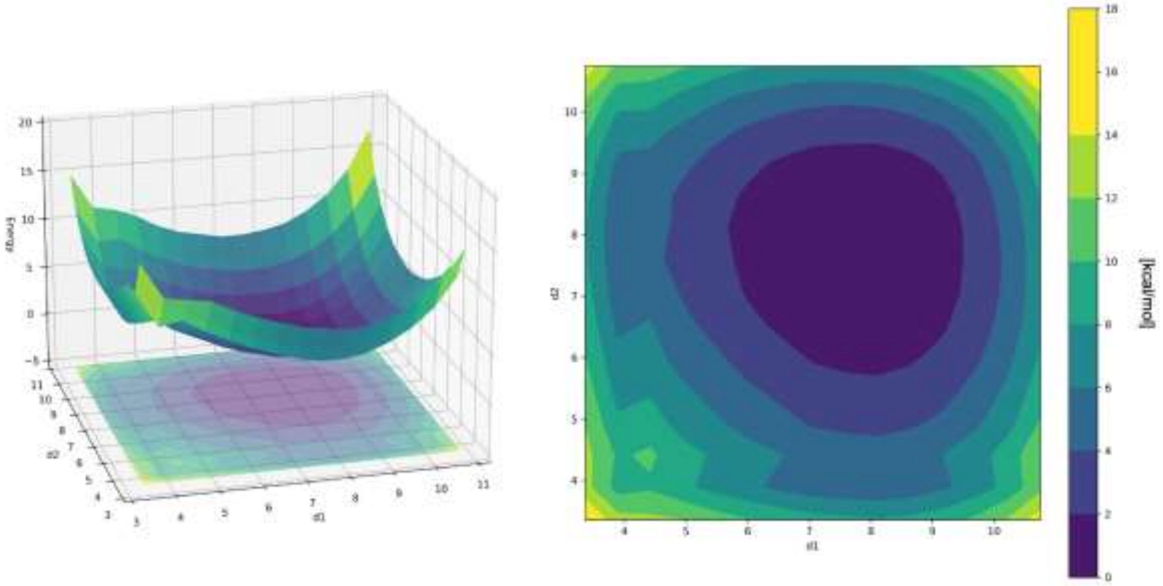

Figure S6. 3D plot (left) and heatmap (right) of DFT relaxed energy scans of *endo*-**N** conformers obtained from xTB showing the stability of the observed conformers corresponding to the distance (d1, d2) of adjacent ligands. DFT and xTB scans were performed in DMSO.

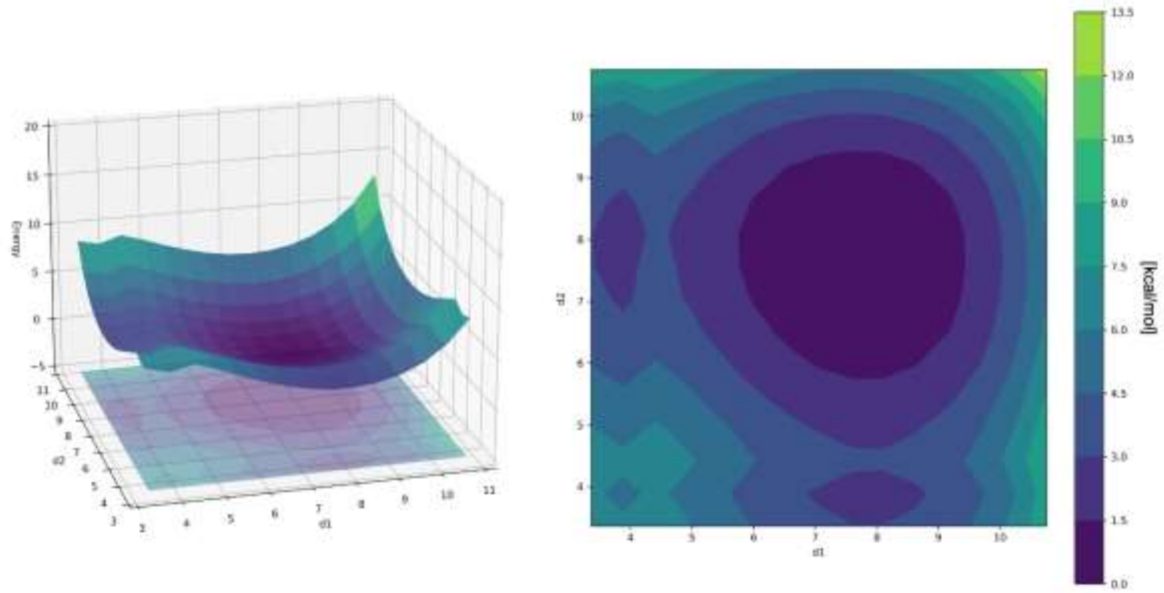

Figure S7. 3D plot (left) and heatmap (right) of DFT relaxed energy scans of *endo*-**N** conformers obtained from xTB showing the stability of the observed conformers corresponding to the distance (d1, d2) of adjacent ligands. DFT and xTB scans were performed in water.

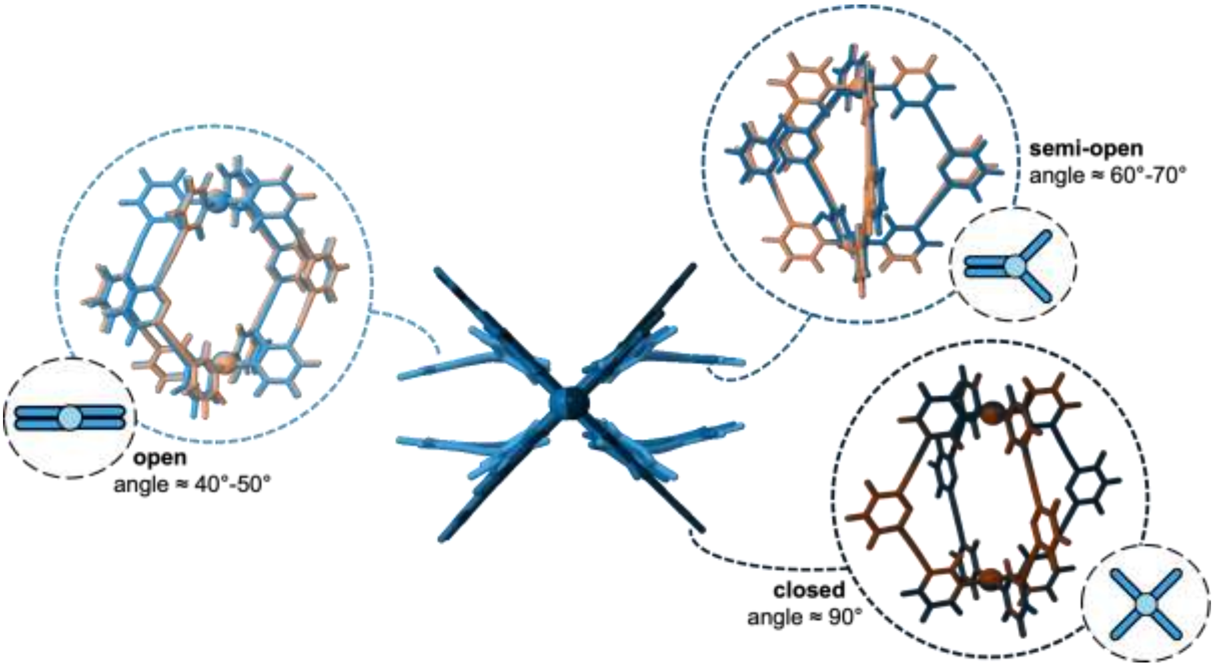

Figure S8. Overlay of optimized structures of the three conformations extracted from MD simulations for the *endo*-**N** cage. For all three conformations, open (top left), semi-open (top right) and closed (bottom right), extracted snapshots are overlaid from simulations in both solvents, water (blue colors) and DMSO (orange colors).

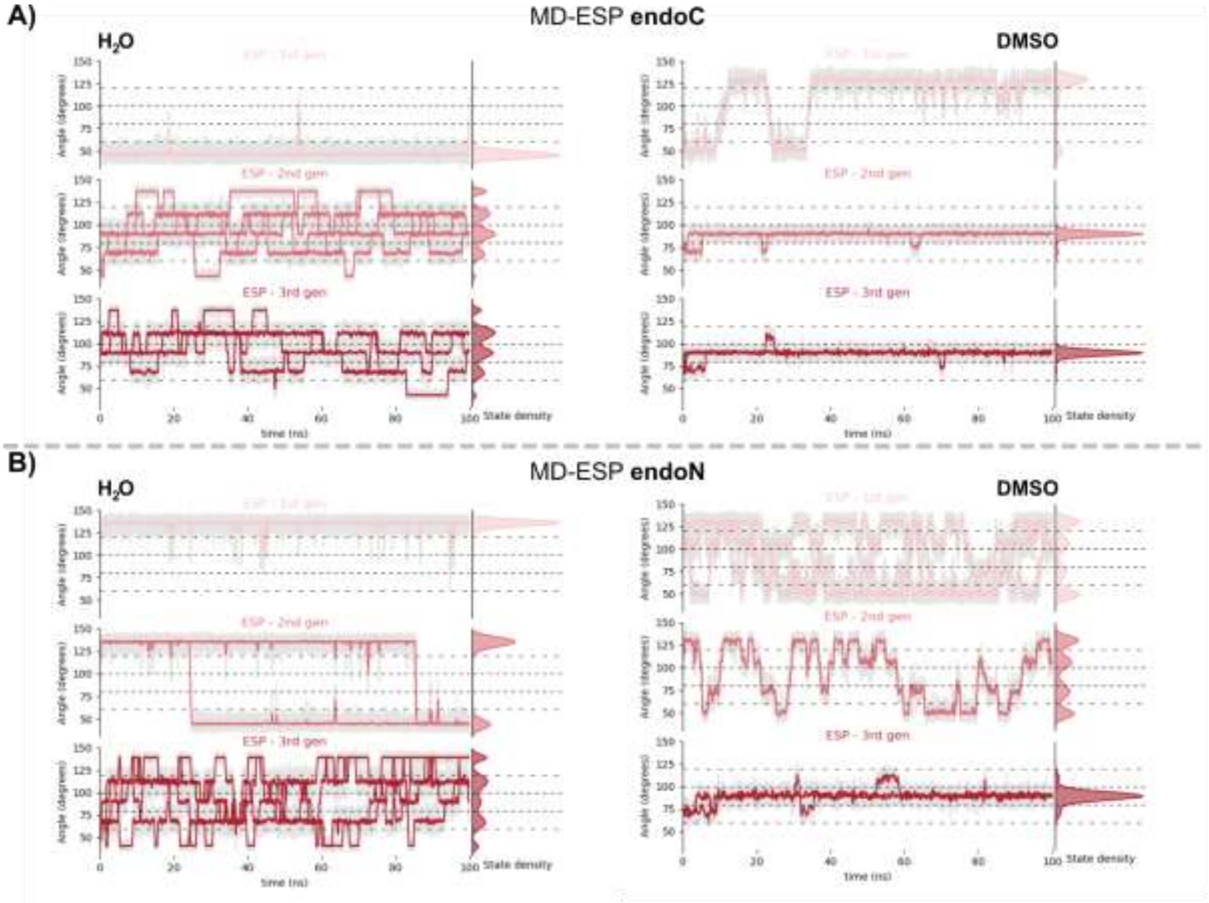

Figure S9. Comparison of the conformational changes observed over time during MD as a function of the angle between two adjacent organic ligands for *endo*-**C** (**A**) and *endo*-**N** (**B**) MCgs using classical FF. Analyses were performed for the three generations of force fields fitted with ESP partial charges for both solvents, water (left) and DMSO (right), respectively. For each force field generation, four replicas of MD simulations were conducted, all contributing to the displayed distributions.

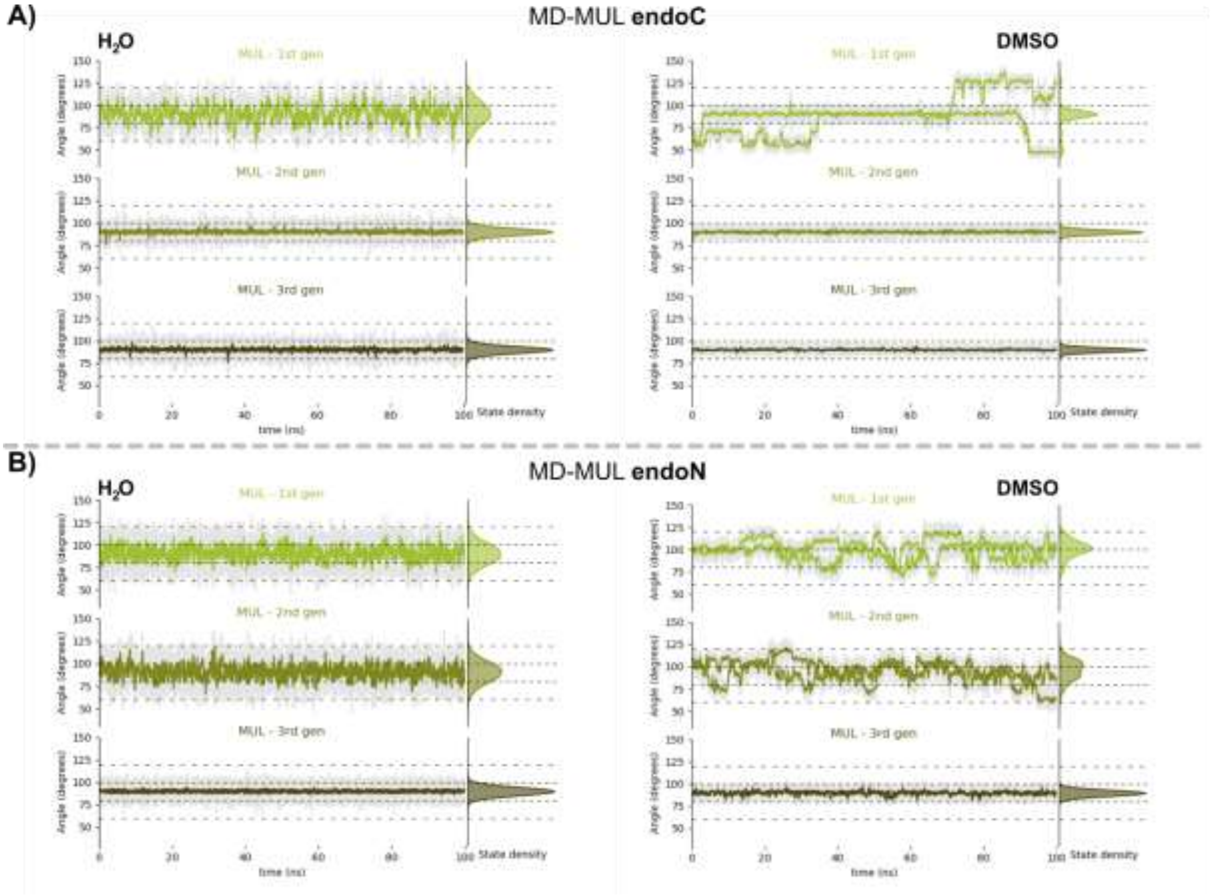

Figure S10. Comparison of the conformational changes observed over time during MD as a function of the angle between two adjacent organic ligands for *endo*-**C** (**A**) and *endo*-**N** (**B**) MCgs using classical FF. Analyses were performed for the three generations of force fields fitted with MUL partial charges for both solvents, water (left) and DMSO (right), respectively. For each force field generation, four replicas of MD simulations were conducted, all contributing to the displayed distributions.

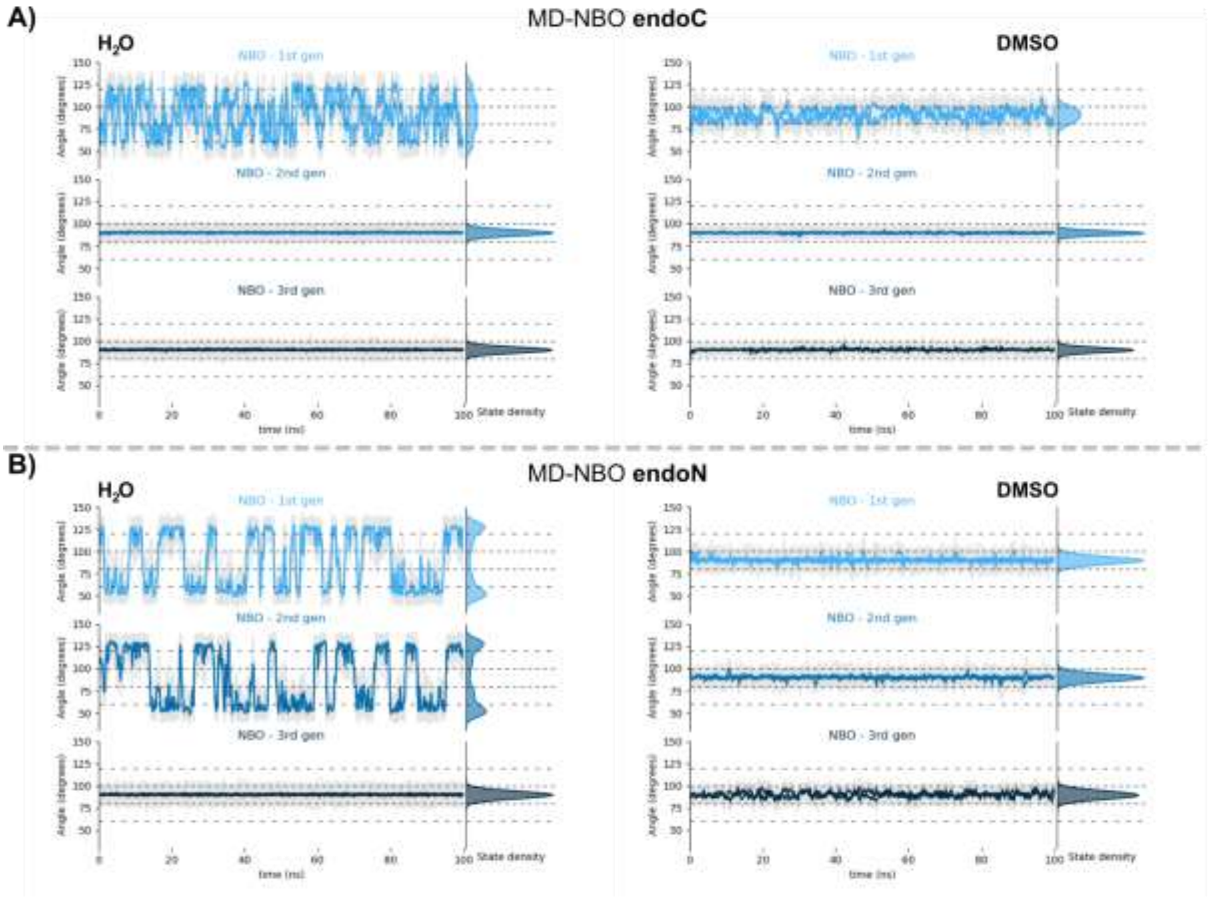

Figure S11. Comparison of the conformational changes observed over time during MD as a function of the angle between two adjacent organic ligands for *endo*-**C** (**A**) and *endo*-**N** (**B**) MCgs using classical FF. Analyses were performed for the three generations of force fields fitted with NBO partial charges for both solvents, water (left) and DMSO (right), respectively. For each force field generation, four replicas of MD simulations were conducted, all contributing to the displayed distributions.

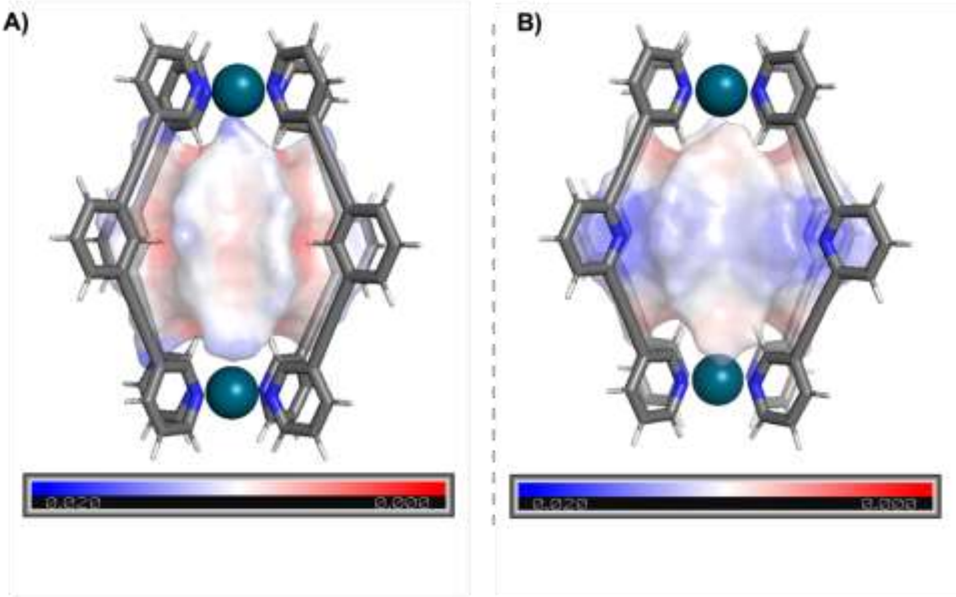

Figure S12. Representation of hydrophobicity and hydrophilicity (Å^3) in the MCg cavities of: **A)** the *endo*-**C** cage, and **B)** of the *endo*-**N** cage. Hydrophobic areas are highlighted in red, while the hydrophilic ones in blue. The areas were calculated using the *cagecavitycalc*[49] tool.

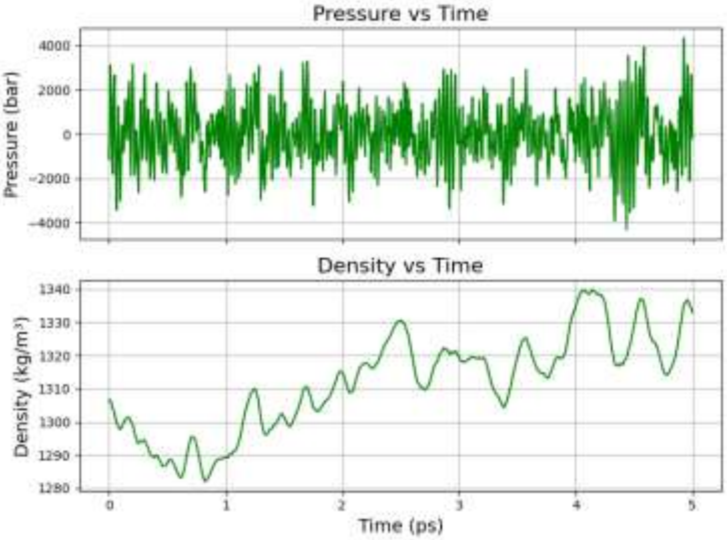

Figure S13. Representative equilibration of system pressure (top) and density (bottom), prior to simulation run productions of the *endo*-**C** in DMSO, using MLIP v2. All other setups for the MLIPs reported in this paper were equilibrated in the same way.

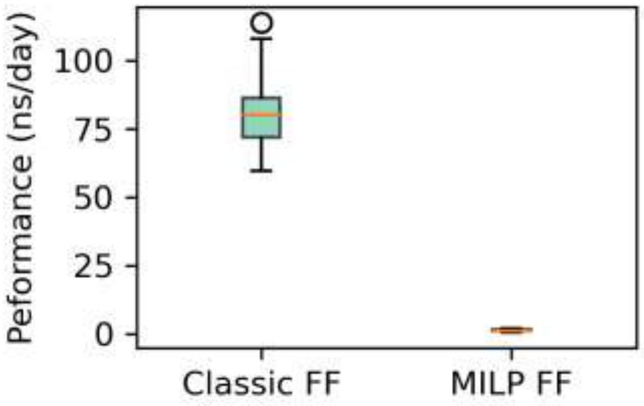

Figure S14. Box plot for graphical comparison of simulation performance of classical methods (left) versus MLIP-based MD simulations (right).

List of Included Files

All files listed below can be found in the respective folder in the Zenodo repository (https://zenodo.org/records/16874121).

Scripts S1 - stk script for MCg construction

Files S1 - Scan molecular structures

Files S2 - Force Field parameters of all force field generations and charge models

Files S3 - Files of raw and averaged atomic partial charges of all charge models

Files S4 - MD setup files

Files S5 - MD input files and initial structures

Files S6 - MD trajectories for visualization (25 ns excerpts in water or DMSO using *3rd* generation FFs with RESP charges, rendered using VMD[48])

Files S7 - MLIP training files

Files S8 - MLIP MD configuration and input files.

REFERENCES

(1) Turcani, L.; Tarzia, A.; Szczypiński, F. T.; Jelfs, K. E. *Stk* : An Extendable Python Framework for Automated Molecular and Supramolecular Structure Assembly and Discovery. *J. Chem. Phys.* **2021**, *154* (21), 214102. https://doi.org/10.1063/5.0049708.

(2) Tarzia, A. MCHammer. https://github.com/andrewtarzia/MCHammer.

(3) Neese, F. The ORCA Program System. *WIREs Comput. Mol. Sci.* **2012**, *2* (1), 73–78. https://doi.org/10.1002/wcms.81.

(4) Bannwarth, C.; Ehlert, S.; Grimme, S. GFN2-xTB—An Accurate and Broadly Parametrized Self-Consistent Tight-Binding Quantum Chemical Method with Multipole Electrostatics and Density-Dependent Dispersion Contributions. *J. Chem. Theory Comput.* **2019**, *15* (3), 1652–1671. https://doi.org/10.1021/acs.jctc.8b01176.

(5) Ehlert, S.; Stahn, M.; Spicher, S.; Grimme, S. Robust and Efficient Implicit Solvation Model for Fast Semiempirical Methods. *J. Chem. Theory Comput.* **2021**, *17* (7), 4250–4261. https://doi.org/10.1021/acs.jctc.1c00471.

(6) Perdew, J. P.; Burke, K.; Ernzerhof, M. Generalized Gradient Approximation Made Simple. *Phys. Rev. Lett.* **1996**, *77* (18), 3865–3868. https://doi.org/10.1103/PhysRevLett.77.3865.

(7) Perdew, J. P.; Burke, K.; Ernzerhof, M. Generalized Gradient Approximation Made Simple [Phys. Rev. Lett. 77, 3865 (1996)]. *Phys. Rev. Lett.* **1997**, *78* (7), 1396–1396. https://doi.org/10.1103/PhysRevLett.78.1396.

(8) Grimme, S.; Hansen, A.; Brandenburg, J. G.; Bannwarth, C. Dispersion-Corrected Mean-Field Electronic Structure Methods. *Chem. Rev.* **2016**, *116* (9), 5105–5154. https://doi.org/10.1021/acs.chemrev.5b00533.

(9) Weigend, F.; Ahlrichs, R. Balanced Basis Sets of Split Valence, Triple Zeta Valence and Quadruple Zeta Valence Quality for H to Rn: Design and Assessment of Accuracy. *Phys. Chem. Chem. Phys.* **2005**, *7* (18), 3297. https://doi.org/10.1039/b508541a.

(10) Marenich, A. V.; Cramer, C. J.; Truhlar, D. G. Universal Solvation Model Based on Solute Electron Density and on a Continuum Model of the Solvent Defined by the Bulk Dielectric Constant and Atomic Surface Tensions. *J. Phys. Chem. B* **2009**, *113* (18), 6378–6396. https://doi.org/10.1021/jp810292n.

(11) Seminario, J. M. Calculation of Intramolecular Force Fields from Second-Derivative Tensors. *Int. J. Quantum Chem.* **1996**, *60* (7), 1271–1277. https://doi.org/10.1002/(SICI)1097-461X(1996)60:7<1271::AID-QUA8>3.0.CO;2-W.

(12) Malde, A. K.; Zuo, L.; Breeze, M.; Stroet, M.; Poger, D.; Nair, P. C.; Oostenbrink, C.; Mark, A. E. An Automated Force Field Topology Builder (ATB) and Repository: Version 1.0. *J. Chem. Theory Comput.* **2011**, *7* (12), 4026–4037. https://doi.org/10.1021/ct200196m.

(13) Li, P.; Roberts, B. P.; Chakravorty, D. K.; Merz, K. M. Rational Design of Particle Mesh Ewald Compatible Lennard-Jones Parameters for +2 Metal Cations in Explicit Solvent. *J. Chem. Theory Comput.* **2013**, *9* (6), 2733–2748. https://doi.org/10.1021/ct400146w.

(14) Piskorz, T. K.; Lee, B.; Zhan, S.; Duarte, F. Metallicious: Automated Force-Field Parametrization of Covalently Bound Metals for Supramolecular Structures. June 14, 2024. https://doi.org/10.26434/chemrxiv-2024-383j5.

(15) Boaler, P. J.; Piskorz, T. K.; Bickerton, L. E.; Wang, J.; Duarte, F.; Lloyd-Jones, G. C.; Lusby, P. J. Origins of High-Activity Cage-Catalyzed Michael Addition. *J. Am. Chem. Soc.* **2024**, *146* (28), 19317–19326. https://doi.org/10.1021/jacs.4c05160.

(16) Brammer, J. C.; Blanke, G.; Kellner, C.; Hoffmann, A.; Herres-Pawlis, S.; Schatzschneider, U. TUCAN: A Molecular Identifier and Descriptor Applicable to the Whole Periodic Table from Hydrogen to Oganesson. *J. Cheminformatics* **2022**, *14* (1), 66. https://doi.org/10.1186/s13321-022-00640-5.

(17) Gaussian 16, Revision C.01, M. J. Frisch, G. W. Trucks, H. B. Schlegel, G. E. Scuseria, M. A. Robb, J. R. Cheeseman, G. Scalmani, V. Barone, G. A. Petersson, H. Nakatsuji, X. Li, M. Caricato, A. V. Marenich, J. Bloino, B. G. Janesko, R. Gomperts, B. Mennucci, H. P. Hratchian, J. V. Ortiz, A. F. Izmaylov, J. L. Sonnenberg, D. Williams-Young, F. Ding, F. Lipparini, F. Egidi, J. Goings, B. Peng, A. Petrone, T. Henderson, D. Ranasinghe, V. G. Zakrzewski, J. Gao, N. Rega, G. Zheng, W. Liang, M. Hada, M. Ehara, K. Toyota, R. Fukuda, J. Hasegawa, M. Ishida, T. Nakajima, Y. Honda, O. Kitao, H. Nakai, T. Vreven, K. Throssell, J. A. Montgomery, Jr., J. E. Peralta, F. Ogliaro, M. J. Bearpark, J. J. Heyd, E. N. Brothers, K. N. Kudin, V. N. Staroverov, T. A. Keith, R. Kobayashi, J. Normand, K. Raghavachari, A. P. Rendell, J. C. Burant, S. S. Iyengar, J. Tomasi, M. Cossi, J. M. Millam, M. Klene, C. Adamo, R. Cammi, J. W. Ochterski, R. L. Martin,

K. Morokuma, O. Farkas, J. B. Foresman, and D. J. Fox, Gaussian, Inc., Wallingford CT, 2016. 2016.
(18) Glendening, E. D.; Landis, C. R.; Weinhold, F. *NBO 7.0* : New Vistas in Localized and Delocalized Chemical Bonding Theory. *J. Comput. Chem.* **2019**, *40* (25), 2234–2241. https://doi.org/10.1002/jcc.25873.
(19) *The Structure of Small Molecules and Ions*; Naaman, R., Vager, Z., Eds.; Springer US: Boston, MA, 1988. https://doi.org/10.1007/978-1-4684-7424-4.
(20) Becke, A. D. Density-Functional Thermochemistry. III. The Role of Exact Exchange. *J. Chem. Phys.* **1993**, *98* (7), 5648–5652. https://doi.org/10.1063/1.464913.
(21) Lee, C.; Yang, W.; Parr, R. G. Development of the Colle-Salvetti Correlation-Energy Formula into a Functional of the Electron Density. *Phys. Rev. B* **1988**, *37* (2), 785–789. https://doi.org/10.1103/PhysRevB.37.785.
(22) Vosko, S. H.; Wilk, L.; Nusair, M. Accurate Spin-Dependent Electron Liquid Correlation Energies for Local Spin Density Calculations: A Critical Analysis. *Can. J. Phys.* **1980**, *58* (8), 1200–1211. https://doi.org/10.1139/p80-159.
(23) Stephens, P. J.; Devlin, F. J.; Chabalowski, C. F.; Frisch, M. J. Ab Initio Calculation of Vibrational Absorption and Circular Dichroism Spectra Using Density Functional Force Fields. *J. Phys. Chem.* **1994**, *98* (45), 11623–11627. https://doi.org/10.1021/j100096a001.
(24) Ditchfield, R.; Hehre, W. J.; Pople, J. A. Self-Consistent Molecular-Orbital Methods. IX. An Extended Gaussian-Type Basis for Molecular-Orbital Studies of Organic Molecules. *J. Chem. Phys.* **1971**, *54* (2), 724–728. https://doi.org/10.1063/1.1674902.
(25) Wadt, W. R.; Hay, P. J. *Ab Initio* Effective Core Potentials for Molecular Calculations. Potentials for Main Group Elements Na to Bi. *J. Chem. Phys.* **1985**, *82* (1), 284–298. https://doi.org/10.1063/1.448800.
(26) Hay, P. J.; Wadt, W. R. *Ab Initio* Effective Core Potentials for Molecular Calculations. Potentials for the Transition Metal Atoms Sc to Hg. *J. Chem. Phys.* **1985**, *82* (1), 270–283. https://doi.org/10.1063/1.448799.
(27) Hay, P. J.; Wadt, W. R. *Ab Initio* Effective Core Potentials for Molecular Calculations. Potentials for K to Au Including the Outermost Core Orbitals. *J. Chem. Phys.* **1985**, *82* (1), 299–310. https://doi.org/10.1063/1.448975.
(28) Dunning Jr., T. H.; Hay, P. J. *Methods of Electronic Structure Theory*; Schaefer III, H. F., Ed.; Plenum Press, 1977; Vol. 2.
(29) Mulliken, R. S. Electronic Population Analysis on LCAO–MO Molecular Wave Functions. I. *J. Chem. Phys.* **1955**, *23* (10), 1833–1840. https://doi.org/10.1063/1.1740588.
(30) Csizmadia, I. G. *Theory and Practice of MO Calculations on Organic Molecules*, 1. Aufl.; Elsevier Reference Monographs: s.l., 1976.
(31) Kühne, T. D.; Iannuzzi, M.; Del Ben, M.; Rybkin, V. V.; Seewald, P.; Stein, F.; Laino, T.; Khaliullin, R. Z.; Schütt, O.; Schiffmann, F.; Golze, D.; Wilhelm, J.; Chulkov, S.; Bani-Hashemian, M. H.; Weber, V.; Borštnik, U.; Taillefumier, M.; Jakobovits, A. S.; Lazzaro, A.; Pabst, H.; Müller, T.; Schade, R.; Guidon, M.; Andermatt, S.; Holmberg, N.; Schenter, G. K.; Hehn, A.; Bussy, A.; Belleflamme, F.; Tabacchi, G.; Glöß, A.; Lass, M.; Bethune, I.; Mundy, C. J.; Plessl, C.; Watkins, M.; VandeVondele, J.; Krack, M.; Hutter, J. CP2K: An Electronic Structure and Molecular Dynamics Software Package - Quickstep: Efficient and Accurate Electronic Structure Calculations. *J. Chem. Phys.* **2020**, *152* (19), 194103. https://doi.org/10.1063/5.0007045.
(32) Adamo, C.; Barone, V. Toward Reliable Density Functional Methods without Adjustable Parameters: The PBE0 Model. *J. Chem. Phys.* **1999**, *110* (13), 6158–6170. https://doi.org/10.1063/1.478522.
(33) Chen, D.-L.; Stern, A. C.; Space, B.; Johnson, J. K. Atomic Charges Derived from Electrostatic Potentials for Molecular and Periodic Systems. *J. Phys. Chem. A* **2010**, *114* (37), 10225–10233. https://doi.org/10.1021/jp103944q.
(34) Bochevarov, A. D.; Harder, E.; Hughes, T. F.; Greenwood, J. R.; Braden, D. A.; Philipp, D. M.; Rinaldo, D.; Halls, M. D.; Zhang, J.; Friesner, R. A. Jaguar: A High- performance Quantum Chemistry Software Program with Strengths in Life and Materials Sciences. *Int. J. Quantum Chem.* **2013**, *113* (18), 2110–2142. https://doi.org/10.1002/qua.24481.
(35) Bayly, C. I.; Cieplak, P.; Cornell, W.; Kollman, P. A. A Well-Behaved Electrostatic Potential Based Method Using Charge Restraints for Deriving Atomic Charges: The RESP Model. *J. Phys. Chem.* **1993**, *97* (40), 10269–10280. https://doi.org/10.1021/j100142a004.
(36) Wang, L.; O'Mara, M. L. PsiRESP: Calculating RESP Charges with Psi4. *J. Open Source Softw.* **2022**, *7* (73), 4100. https://doi.org/10.21105/joss.04100.

(37) Abraham, M. J.; Murtola, T.; Schulz, R.; Páll, S.; Smith, J. C.; Hess, B.; Lindahl, E. GROMACS: High Performance Molecular Simulations through Multi-Level Parallelism from Laptops to Supercomputers. *SoftwareX* **2015**, *1–2*, 19–25. https://doi.org/10.1016/j.softx.2015.06.001.
(38) Lindorff- Larsen, K.; Piana, S.; Palmo, K.; Maragakis, P.; Klepeis, J. L.; Dror, R. O.; Shaw, D. E. Improved Side- chain Torsion Potentials for the Amber ff99SB Protein Force Field. *Proteins Struct. Funct. Bioinforma.* **2010**, *78* (8), 1950–1958. https://doi.org/10.1002/prot.22711.
(39) Hess, B.; Bekker, H.; Berendsen, H. J. C.; Fraaije, J. G. E. M. LINCS: A Linear Constraint Solver for Molecular Simulations. *J. Comput. Chem.* **1997**, *18* (12), 1463–1472. https://doi.org/10.1002/(SICI)1096-987X(199709)18:12<1463::AID-JCC4>3.0.CO;2-H.
(40) Nosé, S. A Molecular Dynamics Method for Simulations in the Canonical Ensemble. *Mol. Phys.* **1984**, *52* (2), 255–268. https://doi.org/10.1080/00268978400101201.
(41) Parrinello, M.; Rahman, A. Crystal Structure and Pair Potentials: A Molecular-Dynamics Study. *Phys. Rev. Lett.* **1980**, *45* (14), 1196–1199. https://doi.org/10.1103/PhysRevLett.45.1196.
(42) Paszke, A.; Gross, S.; Massa, F.; Lerer, A.; Bradbury, J.; Chanan, G.; Killeen, T.; Lin, Z.; Gimelshein, N.; Antiga, L.; Desmaison, A.; Köpf, A.; Yang, E.; DeVito, Z.; Raison, M.; Tejani, A.; Chilamkurthy, S.; Steiner, B.; Fang, L.; Bai, J.; Chintala, S. PyTorch: An Imperative Style, High-Performance Deep Learning Library. arXiv 2019. https://doi.org/10.48550/ARXIV.1912.01703.
(43) VandeVondele, J.; Krack, M.; Mohamed, F.; Parrinello, M.; Chassaing, T.; Hutter, J. Quickstep: Fast and Accurate Density Functional Calculations Using a Mixed Gaussian and Plane Waves Approach. *Comput. Phys. Commun.* **2005**, *167* (2), 103–128. https://doi.org/10.1016/j.cpc.2004.12.014.
(44) Batzner, S.; Musaelian, A.; Sun, L.; Geiger, M.; Mailoa, J. P.; Kornbluth, M.; Molinari, N.; Smidt, T. E.; Kozinsky, B. E(3)-Equivariant Graph Neural Networks for Data-Efficient and Accurate Interatomic Potentials. *Nat. Commun.* **2022**, *13* (1), 2453. https://doi.org/10.1038/s41467-022-29939-5.
(45) Thompson, A. P.; Aktulga, H. M.; Berger, R.; Bolintineanu, D. S.; Brown, W. M.; Crozier, P. S.; In 'T Veld, P. J.; Kohlmeyer, A.; Moore, S. G.; Nguyen, T. D.; Shan, R.; Stevens, M. J.; Tranchida, J.; Trott, C.; Plimpton, S. J. LAMMPS - a Flexible Simulation Tool for Particle-Based Materials Modeling at the Atomic, Meso, and Continuum Scales. *Comput. Phys. Commun.* **2022**, *271*, 108171. https://doi.org/10.1016/j.cpc.2021.108171.
(46) Izaguirre, J. A.; Catarello, D. P.; Wozniak, J. M.; Skeel, R. D. Langevin Stabilization of Molecular Dynamics. *J. Chem. Phys.* **2001**, *114* (5), 2090–2098. https://doi.org/10.1063/1.1332996.
(47) Jorgensen, W. L.; Chandrasekhar, J.; Madura, J. D.; Impey, R. W.; Klein, M. L. Comparison of Simple Potential Functions for Simulating Liquid Water. *J. Chem. Phys.* **1983**, *79* (2), 926–935. https://doi.org/10.1063/1.445869.
(48) Humphrey, W.; Dalke, A.; Schulten, K. VMD: Visual Molecular Dynamics. *J. Mol. Graph.* **1996**, *14* (1), 33–38. https://doi.org/10.1016/0263-7855(96)00018-5.
(49) Martí-Centelles, V.; Piskorz, T.K.; Duarte, F. CageCavityCalc (*C* 3): A Computational Tool for Calculating and Visualizing Cavities in Molecular Cages. *J. Chem. Inf. Model.* **2024**, *65* (14), 5604-5616. https://doi.org/10.1021/acs.jcim.4c00355.
